# Herbal plants traded at the Kaili medicinal market, Guizhou, China

**DOI:** 10.1186/s13002-021-00495-4

**Published:** 2021-11-29

**Authors:** Sizhao Liu, Beixi Zhang, Jiangju Zhou, Qiyi Lei, Qiong Fang, Edward J. Kennelly, Chunlin Long

**Affiliations:** 1 Key Laboratory of Ecology and Environment in Minority Areas, Minzu University of China, National Ethnic Affairs Commission, Beijing, 100081 China; 2 College of Life and Environmental Sciences, Minzu University of China, Beijing, 100081 China; 3 Key Laboratory of Ethnomedicine,, Minzu University of China, Ministry of Education, Beijing, 100081 China; 4 School of Life and Health Science, Kaili University, Guizhou, 556000 China; 5 Lehman College, City University of New York, Bronx, NY 10468 USA; 6 Kunming Institute of Botany, Chinese Academy of Sciences, Kunming, 650201 China

**Keywords:** Miao people, Medicinal markets, Kaili, Miao medicinal plants, Traditional knowledge

## Abstract

**Background:**

Marketplaces reflect not only the commerce of an area, but also its culture. In Qiandongnan Miao and Dong Autonomous Prefecture with Kaili as its capital, Guizhou Province, China, traditional medicine is thriving in both rural and urban areas. The local people rely extensively on plants for traditional medicines, and these are commonly sold in local specialized markets. The Kaili medicinal market is the biggest in the prefecture. However, ethnobotanical study on herbal plants traded in the traditional market in Kaili has not been performed. The aims of this study are: (1) to document medicinal plants traded in the Kaili traditional market and the associated traditional knowledge; and (2) to analyze the level of agreement among vendors in the purported uses of medicinal plants by using informant consensus (FIC) and the fidelity level (FL).

**Methods:**

Market surveys were conducted in 2014–2019 to collect information about medicinal plants and associated traditional knowledge. Information including vernacular names, preparation methods, and plant uses was obtained by interviewing 116 vendors of herbal plants. Specimens of fresh and dried herbs, collected as vouchers, were identified by the authors and other botanists at the Minzu University of China, and deposited in the herbarium at Minzu University of China. The level of agreement among information provided by different vendors was assessed using the FIC, and the percentage of vendors claiming the use of a certain medicinal plant for the same indication was assessed with the FL.

**Results:**

The Miao people comprise 53.4% of all informants in this study of medicinal plants. In total, 237 medicinal plant species traded in the Kaili traditional market were recorded. They belong to 219 genera and 107 families. These plants have been categorized into their purported treatments for 20 medical conditions. The inflammation category showed the highest FIC value of 0.95, showing the best agreement among market vendors claiming its usefulness to treat this condition. The FL index helped to identify 15 culturally important medicinal plant species based on the reported uses by 20 or more vendors in the market. Three medicinal plant species, *Eleutherococcus gracilistylus, Sargentodoxa cuneata*, and *Stephania cepharantha*, had an FL > 90%, being used to treat sprains/traumas, rheumatism, and heat/toxins.

**Conclusions:**

The medicinal plants sold in the Kaili market are highly diverse and have unique medicinal characteristics. The Miao people often use traditional herbal plants for disease prevention and thereby prioritize the use of medicinal plants in everyday life. The future of this medicinal marketplace, however, is uncertain since few young people (< 30 years old) are vendors or customers. Therefore, it is urgent to conserve traditional ethnomedicinal culture in local communities and pass on the associated traditional knowledge to future generations in this prefecture. And the next step should include further studies on FL > 90% plants’ chemistry, pharmacology, biological activity, and toxicity for potentially developing functional foods or pharmaceutical products.

## Background

The use of plants for medical treatment and therapy is a practice as old as humanity, dating as far back as the oldest known written documents and found in nearly every known culture [1–3]. Approximately 80% of the world’s population currently use traditional herbal medicines [4–6], and a large number of ethnic medicinal plants are used as raw materials in the pharmaceutical industry. Therefore, millions of people rely on medicinal plants not only for primary health care but also for their livelihood. For example, according to the Guizhou provincial government, the market for Miao medicinal products has doubled in the last 5 years to over 20 billion RMB ($2.95 billion in USD), exceeding the total sum of the Tibetan, Uygur, and Mongolian medicines [7]. Therefore, traditional medicinal plants provide valuable information for the synthesis of new drugs and play an important role in modern society.

Traditional markets around the world are known for the trade of plants, minerals, and animals, and regional trade represents an important expression of culture [8–11]. And trade of these products also has been the backbone of the economy in many rural areas, most of which consist of wild harvested goods [12]. In recent years, some ethnobotanical research on traditional markets had been conducted in China, including those in Bijie [13], Jingxi [14], Yangchun [15], Gongcheng [16], Dechang [17], Jianghua [18, 19], Zhenfeng, and Xingren [20]. These studies have contributed to the understanding of plant diversity involved in the trade of medicinal plant species [21, 22].

The Qiandongnan Miao and Dong Autonomous Prefecture is an area with rich biological and cultural diversity in Guizhou Province, Southwest China. It is also well known for its unique karst topography with elevations from 137 to 2178 m above sea level and remarkable vertical climate stratification. There are more than ten ethnic groups native to Qiandongnan Prefecture, making it an ethnic minority group hot spot.

The Miao people live primarily in southern China’s mountainous areas, including Guizhou, Yunnan, Hunan, Guangxi, Chongqing, Sichuan, Hubei, Guangdong, and Hainan. The Qiandongnan Miao and Dong Autonomous Prefecture is the largest Miao community in China, with a population of about 1.86 million, accounting for about 42% of the prefecture’s population [23].

Herbal medicines are an integral part of Miao health and development. Medical clinics in Miao communities are relatively inaccessible and treatments are often costly. The Miao villages are normally surrounded by forests with many medicinal plants, and thus they often use locally sourced herbal medicines. Thus, the Miao people have developed their own traditional medicine with associated indigenous knowledge. In the past decades, some publications have documented Miao medicinal research achievements [24–26]. The Miao medicine is a highly regarded discipline in China and is becoming increasingly popularized in the country.

However, with the rapid development of the Miao medicinal industry, the traditional markets are rapidly decreasing because of the emerging e-trade systems in China and the growth of mini-supermarkets and shops throughout the countryside, but some Miao people in Kaili still keep the custom of trading medicinal plants. Every week, the Miao people who generally live more than 30 km far from urban areas bring medicinal plants to trade in the Kaili medicinal market. It is not easy to conserve the traditional medicinal knowledge maintained by a small population. Although this marketplace is large in scale, it has not been investigated ethnobotanically. It is therefore urgent to document these medicinal plants and the associated traditional knowledge of the Miao people. Research regarding traditional marketplaces can help producers, sellers, healers, and consumers develop an ongoing relationship through knowledge-based supply and demand of medicinal plants and their derivatives.

Therefore, our study focuses on this understudied medicinal marketplace, which reflects the diversity of medicinal plants in the prefecture. There are two primary aims of this study: (1) to record the current use of medicinal plants in Kaili market and associated traditional knowledge; and (2) to explore connections between medicinal plants and vendors using the method of informant consensus (FIC) and to determine the most frequently sold medicinal plant species using the method of fidelity level (FL). Through our study, we try to provide policymakers, researchers, and local people with the necessary information and data for the conservation and sustainable use of traditional Miao medicinal plants and associated traditional knowledge. Furthermore, this study may provide valuable information for future development and also give comprehensive and scientific guidance for local people to consume medicinal herbs more safely.

## Methods

### Study site

**Fig. 1:**
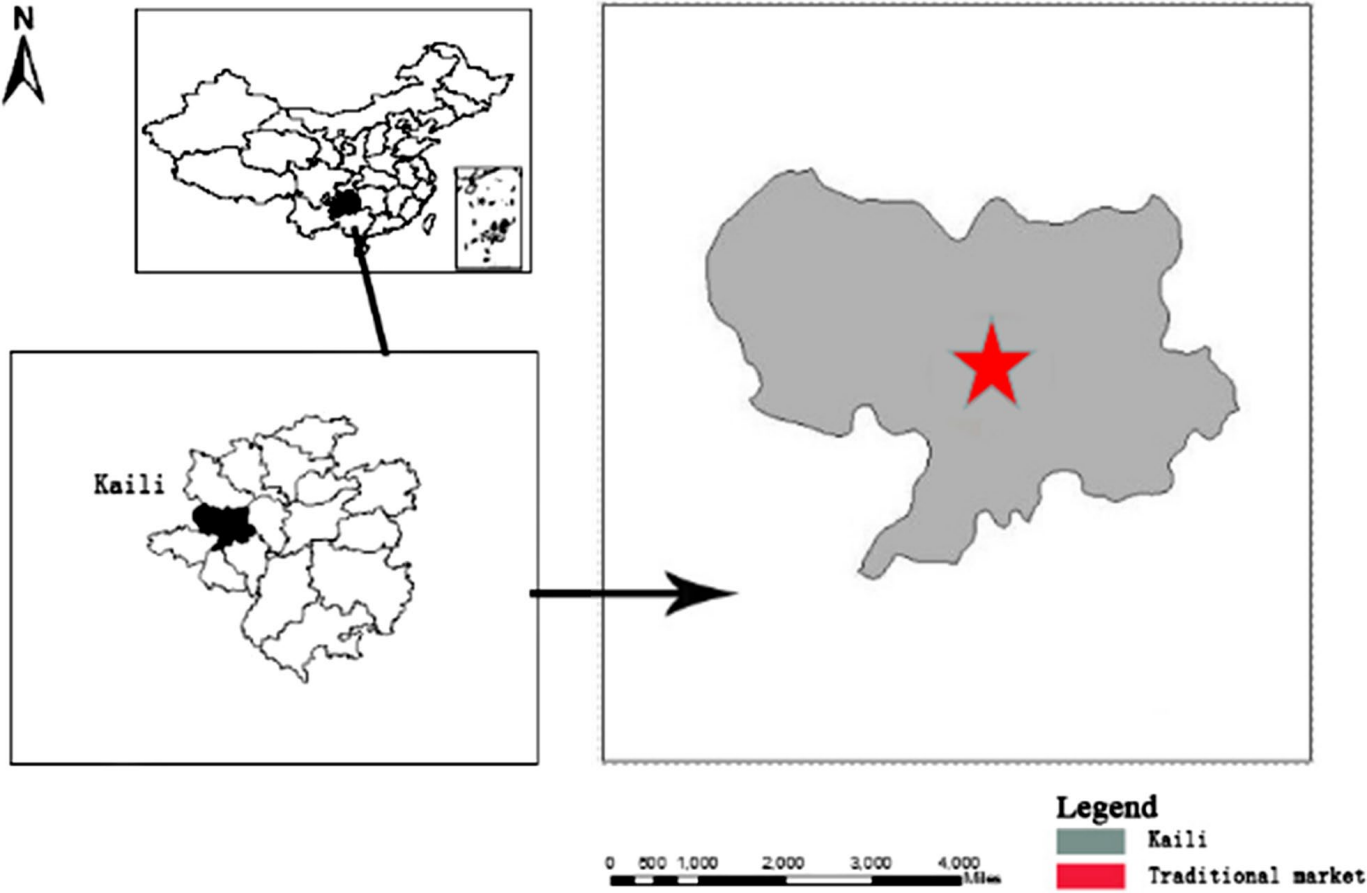
Location of Kaili, the study area in Guizhou

Kaili is the capital of the Qiandongnan Miao and Dong Autonomous Prefecture, located in southeast Guizhou (Fig. 1) at nearly 850 m above sea level. The population of Kaili is dominated by the Miao people who comprise about 63% of the total population. There are dozens of rivers in Kaili that flow into the Yuanjiang River, a branch of Yangtze. The sinkholes and underground caverns in the area are well developed because of its karst topography.

### Kaili medicinal market

**Fig. 2:**
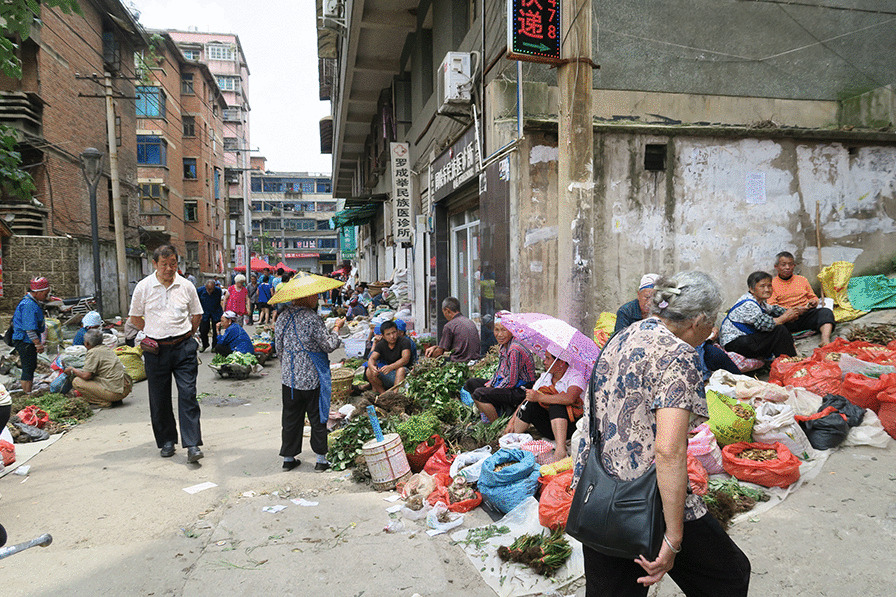
Old site of Kaili traditional medicinal market. Photo by C Long, taken in July 2015

The Kaili medicinal market was founded about 200 years ago by local people, and the trade of medicinal plants was likely developed in conjunction with the sale of other necessities (Fig. 2). In 2016, with the assistance of the local government and urban planners, the market was moved to a new site and developed into a tourist attraction, thereby integrating traditional medicinal culture and economy.

**Fig. 3:**
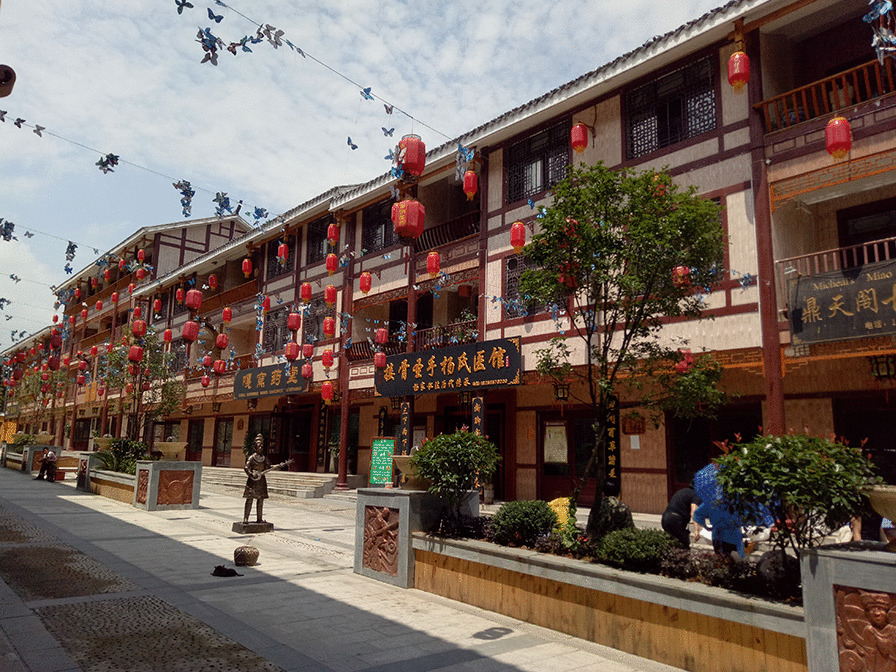
New site of Kaili traditional medicinal market. Photo by S Liu, taken in July 2017

The current market includes two subsectors: traditional medicinal plants and pharmaceutical/ready-to-use-drug markets. The former comprise raw or dried plants with little or no processing. The latter contain processed medicinal plant products (Fig. 3). A variety of participants are involved in the sale of medicinal plants at Kaili traditional market, such as rural harvesters, small retailers, and licensed vendors (Table 1).

**Table 1:** Types of vendors of medicinal plants and definitions of participants within the herbal market

Vendors	Definition
Rural harvesters	Individuals who come from the rural areas surrounding the Kaili, bringing fresh medicinal plants collected by themselves from natural habitats or home gardens. They have good knowledge of Miao medicine
Small retailers	Individuals who occasionally go to Kaili to trade herbal plants to customers, bringing fresh medicinal plants collected by themselves or rural harvesters. They have common knowledge of Miao medicine
Licensed vendors	Individuals who rent a stall in the market import medicinal plants from all over the country. They have poor knowledge of Miao medicine

### Ethnobotanical surveys

**Fig. 4:**
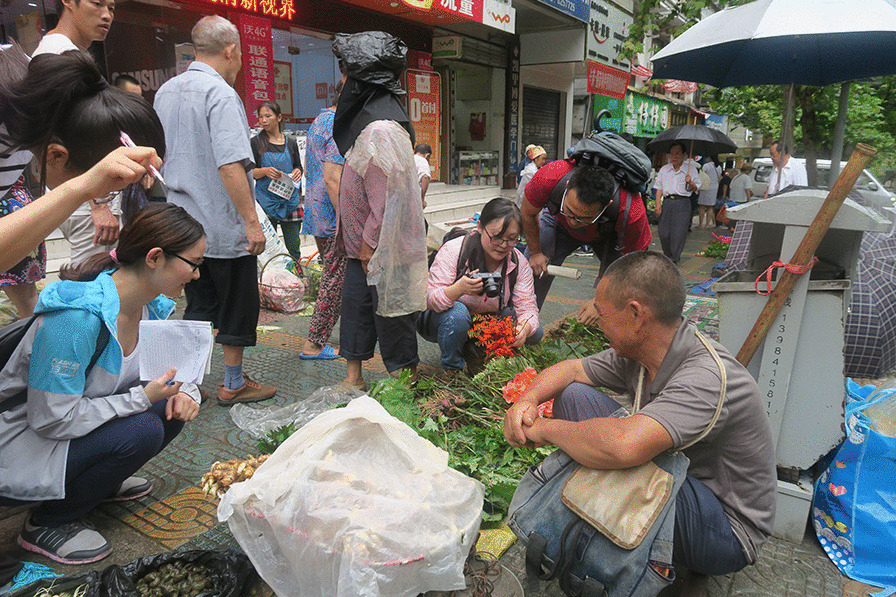
Interviewing in the market. Photo by C Long

A total of 116 vendors (71 male and 45 female) selling medicinal plants in the market were interviewed, ranging in age from 20 to 87 years old, with a mean age of 65. To gather information about medicinal plants in the market, semi-structured interviews with vendors were conducted (Fig. 4). Information from vendors was recorded, including vernacular names of medicinal plants, medicinal uses, parts used, habitat of plants, and therapeutic prescriptions. Eighteen key informants were selected to interview who were either local healers or important custodians and practitioners with rich traditional knowledge of medicinal plants. All these local healers were males.

When interviewing vendors, samples of fresh herbs were purchased at the regular price from each medicinal market as voucher specimens. For the dry herbs sold in the market, we collected specimens from the field, with assistance from local people. Voucher specimens were prepared and deposited in the herbarium at the Minzu University of China in Beijing, China, for future reference. The botanical identities of voucher specimens were confirmed by the authors and other botanists at the Minzu University of China. Plant names were checked with *Flora of China* (http://flora.huh.harvard.edu/china/) and botanical Web sites including http://www.tropicos.org/ and http://www.theplantlist.org.

### Data analysis

The data were summarized using Microsoft Office Excel and organized for statistical analysis. All of the local therapeutic uses of medicinal plants were grouped into 20 medical categories, which were based on the information gathered from the interviewees.

The FIC index was used to measure consensus among vendors regarding the therapeutic use of each medicinal plant [27–29]. The formula is listed as follows:

$${\text{FIC}} = ({\text{Nur}}{-}{\text{Nt}}){/}({\text{Nur}}{-}{1}).$$

Nur refers to the number of therapeutic use reports, grouped in a medical category, from market vendors for a particular medicinal plant, and Nt refers to the total number of medicinal plant species used in a particular medical category. The FIC values range between 0 and 1, where 1 indicates the highest level of market vendor consensus.

The FL index [30] indicates the percentage of vendors claiming the use of a certain medicinal plant for the same therapeutic use, which was grouped in a specific medical category [31–33]. The FL was calculated according to the following formula: FL (%) = (Ip × 100/Iu), where Ip is the number of market vendors who independently claim a therapeutic use of a medicinal plant species to treat a specific illness or disease and Iu is the total number of market vendors that sold the same medicinal plant to treat any given illness or disease.

## Results

### Demographic features of the vendors

A total of 116 medicinal plant vendors (71 male and 45 female) were interviewed at the market. Of these, 62 were Miao people (Table 2). The Miao ethnic people comprised 53.4% of the total interviewees, corresponding with ethnic composition of Qiandongnan Prefecture. The large number of vendors made this market an especially good place to conduct ethnobotanical surveys. We found 50 vendors, ranging in age from 31 to 90, with a median of 60 years old, and few vendors under 30 years old. Most of those younger vendors collect wild medicinal plants and sell them as a part-time activity, while the older generation do this full time. As for the gender structure of the vendors, the number of men and women older than 60 years old was almost the same, while for those under 60 years old, almost twice as many men as women in this group. Most vendors were small retailers, as Table 3 summarizes the number of all the vendors surveyed.

**Table 2:** Demographic profile of the vendors

Gender	Age-group			Linguistic group		
	20–30	31–60	61–90	Miao	Dong	Other
Female	6	17	22	29	8	8
Male	12	33	26	33	20	18
Total	18	50	48	62	28	26

**Table 3:** The numbers of all kinds of vendors surveyed

Vendors	Numbers
Rural harvesters	30
Small retailers	49
Licensed vendors	37

### Miao medicinal plants traded at traditional market

We recorded 237 medicinal plant species traded at the Kaili traditional medicinal market, which were grouped into 219 genera and 107 families. The results provided the following information for each species: scientific name, Chinese name, local name, botanical family, plant part used, disease treated, route of administration, and use value (Table 4). The dominant plant family was Compositae with 23 species (9.6% of the total species), followed by 16 species of Rosaceae (6.7%), 10 species of Labiatae (4.2%), 9 species of Liliaceae (3.8%), while another 63 families were mostly represented by 1 species. Vendors in the marketplace sold different plant parts for the preparation of traditional drugs (e.g., leaves, roots, seeds, barks, and fruits). The whole plants were the most common plant material used, followed by roots. Life forms showed that herbaceous plants constituted the highest proportion with 144 species (60.8%), while there were 47 shrubs (19.8%), 25 trees (10.5%), and 21 lianas (8.9%). The majority of remedies could be prepared from either dried or fresh materials, and some were prepared only from fresh materials, while a few were prepared from dried materials.

**Table 4:** Inventory of medicinal plants traded at the Kaili medicinal market

No.	Scientific name	Chinese name	Miao name	Family	Using part	Preparation method	Use and value	Voucher specimen number
1	*Abelmoschus manihot* (L.) Medicus	Huang shu kui	huangf suf	Malvaceae	Seed, root, flower	Grinding, decoction; pound fresh part applied on the affected area	Promote diuresis; treat strangury; heat-clearing and detoxifying; the blood circulation hematischesis; set a fracture; promote tissue regeneration	KL-139
2	*Acalypha australis* L	Tie xian cai	det nix vud	Euphorbiaceae	Whole plant	Oral, boiled with meat and drunk the soup	Clearing heat and promoting diuresis; cooling blood remove pathogenic heat; disperse accumulations	KL-175
3	*Eleutherococcus nodiflorus* (Ruprecht & Maximowicz) Maximowicz	Ci wu jia	vob bal diangd	Araliaceae	Bark	Oral, boiled with meat and drunk the soup	Strong bones and muscles; expelling wind–damp	KL-053
4	*Achillea wilsoniana* Heimerl ex Hand.-Mazz	Yun nan shi	vob hvid bil	Compositae	Whole plant	Oral, boiled with meat and drunk the soup	Dispelling wind and eliminating dampness; arnica extract; analgesia; detumescence	KL-233
5	*Achyranthes bidentata* Blume	Tu niu xi	jex sangx ghut ngeil niub	Amaranthaceae	Root, dried	Oral and external, boiled with meat and drunk the soup or medicated bath	Promote diuresis; treat strangury; remove urinary calculus; clearing heat and detoxicating; promoting blood circulation to dispel blood stasis	KL-196
6	*Aconitum carmichaeli* Debx	Wu tou	bod jab nangl hlieb	Ranunculaceae	Tuber	Grinding, decoction	Dispelling wind and eliminating dampness; warming womb and channels; eliminating cold stop pain	KL-234
7	*Acorus tatarinowii* Schott	Shi chang pu	jab box vib	Araceae	Rhizome	Oral, grinding, decoction, medicinal liquor	Resolve phlegm; resolving dampness; decreasing swelling to relieving pain	KL-071
8	*Actinidia chinensis* Planch	Mi hou tao	uab mongs dongf	Actinidiaceae	Fruit	Oral	Dry mouth; dyspepsia; anticancer	KL-235
9	*Agrimonia pilosa* Ldb	Long ya cao	jab ghad jil gheib	Rosaceae	Whole plant	Grinding, decoction; boiled with water	Astringency and hemostasis; anti-diarrhea effect; insecticide	KL-236
10	*Adenophora tetraphylla* (Thunb.) Fisch	Lun ye sha shen	ngix gheib ghob	Campanulaceae	Root	Oral, grinding, decoction	Nourishing yin and clearing away heat; moistening lung for removing phlegm; tonifying stomach and promoting fluid	KL-052
11	*Akebia trifoliata* (Thunb.) Koidz	San ye mu tong	zend damgx gir	Lardizabalaceae	Fruit	Oral, boiled with water	Soothing liver and harmonizing stomach; inflammatory swelling	KL-012
12	*Alangium chinense* (Lour.) Harms	Ba jiao feng	ghab jongx deus diek naob dub	Alangiaceae	Root, leaf, stems	Oral and external, grinding and drink with wine	Wind–dampness eliminating; enhance the blood circulation; pain-killing effect	KL-174
13	*Aletris spicata* (Thunb.) Franch	Fen tiao er cai	gad mangl vud	Liliaceae	Root, whole plant	Grinding, decoction	Clearing heat; moistening lung for arresting cough; promoting blood circulation for regulating menstruation; insecticide	KL-183
14	*Alisma orientalis* (Sam.) Juzep	Ze xie	vob gend lix	Alismataceae	Tuber	Taken orally soup	Inducing diuresis and excreting dampness; purge heat; treating stranguria	KL-137
15	*Amorphophallus konjac* C. Koch	Mo yu	jab nangb	Araceae	Tuber	Oral, grinding, decoction, medicinal liquor	Toxic material and removing stasis; resolve phlegm; disperse accumulations; analgesia	KL-011
16	*Ampelopsis delavayana* Planch	San lie ye she pu tao	ghab jongx zend gheid dlub	Vitaceae	Root	Pound fresh part applied on the affected area; medicinal liquor	Promoting blood circulation and removing obstruction in channels	KL-138
17	*Anemone rivularis* Buch.-Ham	Hu zhang cao	zend liul nangb dlub	Ranunculaceae	Whole plant	Pound fresh part applied on the affected area	Heat-clearing and detoxifying; promoting blood flow and tendon relaxation; decreasing swelling to relieving pain	KL-010
18	*Aralia chinensis* L	Song mu	ghab jongx linl det vob hmuk mol	Araliaceae	Root	Oral and external, medicinal liquor, pound fresh part applied on the affected area	Dispelling wind and eliminating dampness; inducing diuresis for removing edema; removing stasis to stop pain	KL-051
19	*Arctium lappa* L	Niu bang	vob dliangb dliek	Compositae	Fruit, root	Oral, boiled with meat and drunk the soup	Wind-heat; promoting eruption; detoxification	KL-172
20	*Ardisia bicolor* Walker	Zi jin niu	jab bib lik jib	Myrsinaceae	Whole plant, dried	Taken orally soup; pound fresh part applied on the affected area	Eliminating phlegm and stopping cough; dampness; promoting blood circulation	KL-195
21	*Ardisia crenata* Sims	Zhu sha gen	jab bik lik jib	Myrsinaceae	Root	Grinding, decoction	Dispelling wind and eliminating dampness; heat-clearing and detoxifying; removing stasis; analgesia	KL-176
22	*Arenaria serpyllifolia* L	Zao zhui	mongb ghait ned	Caryophllaceae	Whole plant	Oral and external, grinding, decoction, medicinal liquor	Heat-clearing and detoxifying; improving eyesight; acute conjunctivitis; hordeolum stye; sore throat	KL-173
23	*Arisaema erubescens* (Wall.) Schott	Yi ba san nan xing	kuad bed vud	Araceae	Tuber	Oral, grinding, decoction	Drying damp and eliminating phlegm; expelling wind and relieving convulsion; detumescence	KL-171
24	*Aristolochia debilis* Sieb. et Zucc.	Ma dou ling	jab mongb qub	Aristolochiaceae	Fruit	Oral, grinding, decoction	Check dysentery; anti-diarrhea effect	KL-111
25	*Artemisia annua* L	Huang hua hao	vob hvid vud	Compositae	Whole plant	Oral and external, pound fresh part applied on the affected area, boiled with water	Clearing summer-heat; preventing further attack of malaria	KL-050
26	*Asarum wulingense* C. F. Liang	Wu ling xi xin	jab niux kab	Aristolochiaceae	Whole plant	Oral, boiled with water, medicinal liquor	Eliminating phlegm and stopping cough; decreasing swelling to relieving pain; dispel wind-cold	KL-072
27	*Asparagus cochinchinensis* (Lour.) Merr	Tian men dong	zend jab ngol hvuk	Liliaceae	Tuber	Grinding, decoction	Nourishing yin and falling fire; clearing lung-heat and moistening dryness	KL-177
28	*Begonia grandis* Dry. subsp. *sinensis* (A. DC.) Irmsch	Zhong hua qiu hai tang	qub haix tangf	Begoniaceae	Tuber	Oral, grinding and drink with wine	Promoting blood circulation for regulating menstruation; hemostasis; check dysentery; postoperative analgesia	KL-009
29	*Belamcanda chinensis* (L.) Redouté	She gan	Vob dak dlangd bad	Iridaceae	Tuber	Oral	Heat-clearing and detoxifying; removing phlegm; activating qi to resolve stagnation	KL-182
30	*Berberis julianae* Schneid	Hao zhu ci	nbox qeub zhent	Berberidaceae	Root, stem, leaf	Oral and external, pound fresh part applied on the affected area, grinding, decoction	Pulmonary tuberculosis; mumps; adenolymphitis; laryngitis; leucorrhea; traumatic injury	KL-008
31	*Berchemia yunnanensis* Franch	Yun nan gou er cha	det nis	Rhamnaceae	Bark	Grinding, decoction	Heat-clearing and detoxifying; treating stranguria to remove dampness; promoting blood circulation and stopping pain; expelling wind and relieving a cough	KL-025
32	*Bidens pilosa* L	San ye gui zhen cao	nangx jub	Compositae	Whole plant	Oral and external, grinding, decoction, medicated bath	Clearing heat and detoxicating; invigorating spleen to remove dampness	KL-136
33	*Bletilla striata* (Thunb. ex Murray) Rchb. F	Bai ji	wus jut	Orchidaceae	Tuber	Grinding, decoction	Astringency and hemostasis; detumescence and promoting granulation;	KL-135
34	*Blumea balsamifera* (L.) DC	Ai na xiang	diangx vob hvid	Compositae	Whole plant	Oral and external, boiled with meat and drunk the soup or medicated bath	Dispelling wind and eliminating dampness; detoxification; promoting blood circulation	KL-007
35	*Boehmeria nivea* (L.) Gaudich	Zhu ma	nos	Urticaceae	Whole plant	Grinding, decoction; taken orally soup; pound fresh part applied on the affected area	Blood cooling and arresting; detoxification; the diuresis detumescence; removing stasis	KL-024
36	*Broussonetia papyrifera* (L.) Vent	Gou shu	det xit hsenb	Moraceae	Fruit	Grinding, decoction	Removing liver fire for improving eyesight; nourishing kidney nourishing yin; lactagogue; invigorating spleen for diuresis	KL-006
37	*Buddleja davidii* Fr	Da ye zui yu cao	nangx dos nail	Buddlejaceae	Root, stems, leaf	External, medicated bath	Relieving rheumatism and cold; invigorating blood circulation and stopping pains	KL-168
38	*Caesalpinia decapetala* (Roth) Alston	Yun shi	ghaob jongx bel jab fab	Leguminosae	Seed	Taken orally soup	Remove coldness; Resolve phlegm to relive cough; dispelling wind and eliminating dampness	KL-178
39	*Callicarpa bodinieri* Levl	Zi zhu	det ghab diod	Verbenaceae	Leaf	Grinding, decoction; pound fresh part applied on the affected area	Hemostasis; decreasing swelling to relieving pain; removing stasis	KL-049
40	*Camellia oleifera* Abel	You cha	det jenl	Theaceae	Fruit	Grinding, decoction; drink with cold water; pound fresh part applied on the affected area	Heat-clearing and detoxifying; Have a laxative effect; insecticide	KL-184
41	*Campanumoea javanica* Bl. subsp. *japonica* (Makino) Hong	Jin qian bao	jab eb wof	Campanulaceae	Root	Oral, grinding, decoction	Moistening lung; engender liquid; hemostasis; lactagogue	KL-197
42	*Canna indica* L	Mei ren jiao	bangx sent hfud	Cannaceae	Tuber	Oral and external, pound fresh part applied on the affected area and grinding, decoction	Heat-clearing and detoxifying; diuresis; regulate the menstrual function; regulating menstruation	KL-181
43	*Cardiospermum halicacabum* L	Dao di ling	geb lieb niongs dab	Sapindaceae	Whole plant	Pound fresh part applied on the affected area	Clearing heat and promoting diuresis; cooling blood remove pathogenic heat	KL-179
44	*Carpesium cernuum* L	Yan guan tou cao	vob yenb	Compositae	Whole plant	Oral, grinding, decoction	Heat-clearing and detoxifying; resolve phlegm; insecticide; hemostasis	KL-005
45	*Carthamus tinctorius* L	Hong hua	bangx xok	Compositae	Flower, dried	Oral, boiled with water	Promoting blood circulation for regulating menstruation; removing stasis and relieving pain	KL-073
46	*Chenopodium ambrosioides* L	Tu jing jie	jab zangd dit	Chenopodiaceae	Whole plant	Oral and external, grinding, decoction, medicated bath	Dispelling wind and eliminating dampness; insecticidal; anti-itch; promoting blood circulation and stopping pain	KL-199
47	*Chimonanthus praecox* (L.) Link	La mei	ghab jongx ghab link det ghab dlub	Calycanthacerae	Root	Oral, grinding, decoction	Relieving rheumatism and cold stopping pains; detoxification	KL-198
48	*Chirita eburnea* Hance	Yan bai cai	ghab naix liod	Gesneriaceae	Whole plant	Oral	Antitussive	KL-185
49	*Chloranthus henryi* Hemsl	Kuan ye jin su lan	jab jex liux	Chloranthaceae	Root	Oral, grinding, decoction	Relaxing tendon and activation collaterals; heat-clearing and detoxifying; decreasing swelling to relieving pain; expelling wind	KL-200
50	*Cibotium barometz* (L.) J. Sm	Jin mao gou	vob yuk jab hlieb	Dicksoniaceae	Tuber	Oral, grinding, decoction	Strengthen the lumbus and knees; expelling wind–damp	KL-180
51	*Cirsium japonicum* DC	Da ji	vob bel bat hlied	Compositae	Root	Oral and external, boiled with meat and drunk the soup or medicated bath	Blood cooling and arresting; detumescence; promoting blood flow	KL-002
52	*Cirsium setosum* (Willd.) MB	Ci er cai	vob bel bat niab	Compositae	Whole plant	Oral and external, boiled with meat and drunk the soup or medicated bath	Blood cooling and arresting; clearing heat for detumescence	KL-074
53	*Clerodendrum bungei* Steud	Chou mu dan	vob hangt ghad	Verbenaceae	Stem, leaf	Grinding, decoction; boiled with meat and drunk the soup	Removing toxicity for detumescence; expelling wind–damp; decreasing blood pressure	KL-169
54	*Clinopodium chinense* (Benth.) O. Ktze	Feng lun cai	jab gangb xongx hlieb	Labiatae	Whole plant	Oral and external, pound fresh part applied on the affected area, boiled with water	Removing toxicity for detumescence; clearing heat; hemostasis	KL-054
55	*Coix lacryma-jobi* L. var. *mayuen* (Roman) Stapf	Yi yi	zend ded	Gramineae	Root	Grinding, decoction	Clearing heat and promoting diuresis; invigorates the spleen and promotes digestion; insecticide	KL-023
56	*Commelina communis* L	Ya tuo cao	vob ghab linx	Commelinaceae	Overground plant	Oral and external, pound fresh part applied on the affected area, boiled with water	Heat-clearing and detoxifying; inducing diuresis for removing edema KL-	KL-075
57	*Coriandrum sativum* L	Yan sui	ghab hlab ngangs caot	Umbelliferae	Aerial part	Taken orally soup; pound fresh part applied on the affected area	Promoting eruption; analgesia; appetizer digestion and detoxification;	KL-201
58	*Coriaria nepalensis* Wall	Ma sang	det wik	Coriariaceae	Root, leaf	External, medicinal liquor or medicated bath	Heat-clearing and detoxifying; detumescence; healing sore and relieving pain; insecticide	KL-170
59	*Cucubalus baccifer* L	Gou jin man	naf roub zhenx hmangb	Caryophyllaceae	Whole plant	Oral and external, grinding, decoction, medicated bath	Expelling wind; disperse accumulations; promoting blood circulation; set a fracture	KL-110
60	*Cucurbita moschata* (Duch. ex Lam.) Duch. ex Poiret	Nan gua	ghab hniub fab diel	Cucurbitaceae	Seed	Oral, grinding, decoction	Insecticide; lactagogue; inducing diuresis for removing edema	KL-001
61	*Cunninghamia lanceolata* (Lamb.) Hook	Sha mu	ghab ot det jib	Taxodiaceae	Bark	Grinding, decoction; medicated bath	Eliminating dampness; detoxification; promoting blood circulation and stopping pain	KL-133
62	*Curculigo orchioides* Gaertn	Xian mao	jab hsod yut	Amaryllidaceae	Tuber	Oral, boiled with meat and drunk the soup	Warm the kidney; strong bones and muscles; dispelling cold and wet	KL-134
63	*Cuscuta japonica* Choisy	Jin deng teng	ghab bas hlat jongb	Convolvulaceae	Seed	Oral, grinding, decoction	Invigorating kidney and nourishing essence; nourishing the liver to improve visual acuity; secure the fetus; anti-diarrhea effect	KL-186
64	*Cyanotis vaga* (Lour.) Schultes. et J. H. Schultes	Lan er cao	laif eex caox	Commelinaceae	Whole plant	Oral and external, grinding, decoction, medicated bath	Expelling wind–damp; relaxing tendon and activation collaterals; diuretic	KL-202
65	*Cynanchum auriculatum* Royle ex Wight	Niu pi xiao	vob bex teb	Asclepiadaceae	Tuber	Oral, boiled with water, medicinal liquor	Improve digestion; replenishing yin and blood; removing toxicity for detumescence	KL-033
66	*Cynoglossum amabile* Stapf et Drumm	Dao ti hu	heb diangd ghod	Boraginaceae	Whole plant	Oral, boiled with meat and drunk the soup	Clearing heat and promoting diuresis; clearing lung and eliminating phlegm; resolve blood stasis and hemostasis	KL-022
67	*Cyperus rotundus* L	Suo cao	nangx songs bat	Cyperaceae	Tuber	Oral and external, pound fresh part applied on the affected area, boiled with water	Regulate the flow of vital energy and remove obstruction toit; regulates menstruation stops pain; anti-abortion mean successful gestation	KL-055
68	*Datura metel* L	Bai hua man tuo luo	jab hmid gangb	Solanaceae	Flower	Grinding, decoction; caution with poison	Antitussive; analgesia	KL-076
69	*Decaisnea insignis* (Griffith) J. D. Hooker et Thomson	Mao er shi	bef ghob ghad	Lardizabalaceae	Root, fruit	Oral and external, pound fresh part applied on the affected area, boiled with water	Antitussive; expelling wind	KL-021
70	*Dendrobium nobile* Lindl	Jin chai shi hu	nangx ghab zat fangx	Orchidaceae	Stem	Grinding, decoction	Nourishing the stomach to improve the production of body fluid; nourishing Yin and clearing heat; tonifying the kidney and improving eyesight	KL-004
71	*Dichondra repens* Forst	Ma ti jin	reib minl zheit	Convolvulaceae	Whole plant	Oral and external, grinding, decoction, medicated bath	Heat-clearing and detoxifying; clearing heat and promoting diuresis	KL-109
72	*Dioscorea bulbifer*a L	Huang du	zend git hsob	Dioscoreaceae	Tuber	Oral, grinding, decoction	Heat-clearing and detoxifying; blood cooling and arresting	KL-003
73	*Diospyros kaki* Thunb	Shi	zend mil	Ebenaceae	Leaf	Oral, boiled with water	Heat-clearing and detoxifying; moistening lung; deficiency of body fluids	KL-056
74	*Dipsacus asperoides* C. Y. Cheng et T. M. Ai	Chuan xu duan	vob qangd niel	Dipsacaceae	Root	Oral and external, pound fresh part applied on the affected area, boiled with water	Strong bones and muscles; nourishing liver and kidney; stanch flooding	KL-112
75	*Drynaria roosii* Nakaike	Hu jue	diangb liox zat	Drynariaceae	Tuber	Oral, boiled with water	Strong bones and muscles; promoting blood circulation and stopping pain;	KL-187
76	*Duchesnea indica* (Andr.) Focke	She mei	bul yuk dax	Rosaceae	Whole plant	Boiled with water; pound fresh part applied on the affected area	Heat-clearing and detoxifying; blood cooling and arresting; antitussive;	KL-032
77	*Dysosma versipellis* (Hance) M. Cheng ex Ying	Ba jiao lian	reib bax gax	Berberidaceae	Rhizome	Oral and external, boiled with water, medicated bath	Removing toxicity for detumescence; insecticide; expelling wind and reducing phlegm;	KL-203
78	*Elaeagnus henryi* Warb. Apud Diels	Yi chang hu tui zi	dhab nex zend jek nangs	Elaeagnaceae	leaf	Oral, boiled with water	Clear the blood and the swelling away; set a fracture to stop pain; calm panting and suppress cough	KL-108
79	*Emilia sonchifolia* (L.) DC	Yi dian hong	vob nab yongd	Compositae	Whole plant	External, medicinal liquor or medicated bath	Diarrhea	KL-013
80	*Epilobium hirsutum* L	Liu ye cai	vob liax lios	Onagraceae	Whole plant	Taken orally soup; pound fresh part applied on the affected area	Heat-clearing and detoxifying; Relieving exterior and promoting dampness; set a fracture; Improve digestion; promoting blood circulation	KL-232
81	*Epimedium acuminatum* Franch	Cu mao yin yang huo	jab ngol xid	Berberidaceae	Whole plant	Oral, boiled with meat and drunk the soup	Reinforcing kidney to strengthen yang; expelling wind–damp	KL-231
82	*Equisetum diffusum* D. Don	Pi san wen jing	nangx diongx nieb	Equisetaceae	Whole plant	Oral, boiled with meat and drunk the soup	Hemostasis; diuretic; improving eyesight	KL-014
83	*Eriobotrya japonica* (Thunb.) Lindl	Pi pa	ghab jongx det zend jab ninx	Rosaceae	Fruit	Boiled with water; taken orally soup	Remove heat from the lung and arrest cough; lactagogue; expelling wind–damp	KL-204
84	*Eucommia ulmoides* Oliver	Du zhong	det dens	Eucommiaceae	Bark	Oral, grinding, decoction	Nourishing liver and kidney; strong bones and muscles; anti-abortion mean successful gestation	KL-107
85	*Eupatorium chinense* L	Hua ze lan	det vit gheib	Compositae	Whole plant	Oral and external, boiled with meat and drunk the soup or medicated bath	Clearing heat and relieving sore throat; cooling blood remove pathogenic heat; eliminating stasis subdue swelling	KL-130
86	*Euphorbia lathylris* L	Xu sui zi	reib lious ros	Euphorbiaceae	Whole plant	Oral, boiled with water	Detoxicating and destroy intestinal worms; relieving water retention with hydragogue; relieving water retention with hydragogue	KL-156
87	*Euphorbia sikkimensis* Boiss	Shui huang hua	jab eb wok	Euphorbiaceae	Root, leaf	External, grinding and drink with wine	Diuretic; heat-clearing and detoxifying	KL-057
88	*Evodia rutaecarpa* (Juss.) Benth	Wu zhu yu	det gaf ved	Rutaceae	Fruit	Taken orally soup	Eliminating cold stop pain; calm the adverse-rising energy; check retching	KL-015
89	*Fallopia multiflora* (Thunb.) Harald	He shou wu	vob hmuk vongx	Polygonaceae	Tuber	Taken orally soup; grinding and drink with wine; pound fresh part applied on the affected area	Ziyin Yangxue; loosening the bowel to relieve constipation; preventing further attack of malaria; expelling wind; detoxification	KL-113
90	*Ficus carica* L	Wu hua guo	ak niangb zend yex	Moraceae	leaf	Taken orally soup; medicated bath	Moistening lung for arresting cough; it is good for spleen; stomach; removing toxicity for detumescence	KL-016
91	*Ficus tikoua* Bur	Di guo	bongt nial tid	Moraceae	Whole plant	Grinding, decoction	Clear away heat and remove dampness; promoting blood circulation to remove meridian obstruction; removing toxicity for detumescence	KL-017
92	*Firmiana platanifolia* (L. f.) Marsili	Wu tong	ghab jongx det hsob nox	Salicaceae	Seed	Grinding, decoction; medicated bath	Strengthen the spleen; regulate qi; aid digestion; hemostasis	KL-018
93	*Foeniculum vulgare* Mill	Hui xiang	xongx hxongb	Umbelliferae	Fruit	Taken orally soup; pound fresh part applied on the affected area	Eliminating cold stop pain	KL-019
94	*Gastrodia elata* Bl	Tian ma	yangf wid vud	Orchidaceae	Tuber	Grinding, decoction	Dizziness; numbness of the limbs; infantile convulsion	KL-020
95	*Gentiana rhodantha* Franch. ex Hemsl	Hong hua long dan	jab juf saix	Gentianaceae	Whole plant	Oral, boiled with water	Heat-clearing and damp-drying drug; detoxification; discharging fire	KL-106
96	*Geranium nepalense* Sweet	Ni bo er lao guan cao	jab ghab ngenx	Geraniaceae	Whole plant	Oral, boiled with meat and drunk the soup	Dispelling wind and eliminating dampness; dredge the meridians and relieve pain; check dysentery; clearing heat	KL-155
97	*Gerbera piloselloides* (L.) Cass	Mao da ding cao	jab bat nex jongx jub	Compositae	Whole plant	Oral, boiled with meat and drunk the soup	Heat-clearing and detoxifying; moistening lung for arresting cough; promoting blood circulation	KL-078
98	*Geum japonicum* Thunb. var. *chinense* F.Bolle	Rou mao lu bian qing	jab heib khob	Rosaceae	Whole plant, dried	Grinding, decoction	Supplementing qi and activating blood circulation; move blood stasis and clear toxins; expelling wind	KL-105
99	*Glechoma longituba* (Nakai) Kupr	Huo xue dan	vob bix seix hlieb	Labiatae	Whole plant	Oral, boiled with meat and drunk the soup	Damp elimination and smoothing showering; heat-clearing and detoxifying; clear the blood and the swelling away; regulate the menstrual function to stop pain	KL-077
100	*Gleditsia sinensis* Lam	Zao jia	bel def def sad bil	Leguminosae	Fruit	Boiled with meat and drunk the soup	Detumescence; insecticide	KL-114
101	*Glochidion puberum* (L.) Hutch	Suan pan zi	zend mil leib	Euphorbiaceae	Root, fruit	Oral, boiled with water	Clearing heat and promoting diuresis; detoxification; promoting blood circulation	KL-058
102	*Gonostegia hirta* (Bl.) Miq	Nuo mi tuan	bas gad nef	Urticaceae	Whole plant	Grinding, decoction;taken orally soup; pound fresh part applied on the affected area; boiled with meat and drunk the soup; medicated bath	Invigorates the spleen and promotes digestion; block blood and break stasis inducing diuresis for removing edema; heat-clearing and detoxifying	KL-079
103	*Grangea maderaspatana* (L.) Poir	Tian ji huang	reib hlol ndenb	Compositae	Whole plant	Oral and external, pound fresh part applied on the affected area, boiled with water	Clearing heat and promoting diuresis; blood-cooling and blood-flow promoting drugs; removing toxicity for detumescence	KL-129
104	*Gynura japonica* (Thunb.) Juel	Ju san qi	jab hsaik laix dliob	Compositae	Root, whole plant	Oral and external, pound fresh part applied on the affected area, boiled with water	Hemostasis; decreasing swelling to relieving pain; heat-clearing and detoxifying	KL-031
105	*Gynostemma pentaphyllum* (Thunb.) Makino	Jiao gu lan	vob ghab did	Cucurbitaceae	Whole plant	Oral and external, pound fresh part applied on the affected area, boiled with water	Heat-clearing and detoxifying; resolve phlegm to relive cough; supplementing qi and nourishing yin; engender liquid; tranquilization	KL-104
106	*Hedera nepalensis* K. Koch	Zhong hua chang chun teng	jab hxend yut	Araliaceae	Whole plant	External, pound fresh part applied on the affected area	Expelling wind and removing toxin; the blood circulation hematischesis; decreasing swelling to relieving pain	KL-154
107	*Hibiscus mutabilis* L	Mu fu rong	det bangx nangl	Malvaceae	Leaf, flower	Pound fresh part applied on the affected area	Heat-clearing and detoxifying; blood cooling and arresting; detumescence; apocenosis	KL-034
108	*Houttuynia cordata* Thunb	Ji cao	vob diuk	Saururaceae	Whole plant	Grinding, decoction	Heat-clearing and detoxifying; drainage of pus and dissolving carbuncle; diuretic and detumescence;	KL-059
109	*Hovenia dulcis* Thunb	Zhi ju	zend ghol bil	Rhamnaceae	Seed, root	Grinding, decoction	Prevent alcoholism; antitussive; check retching; relaxing tendon and activation collaterals	KL-188
110	*Humulus scandens* (Lour.) Merr	Lv cao	bangx nangx lif	Moraceae	Whole plant	Taken orally soup; grinding and drink with wine; pound fresh part applied on the affected area; medicated bath	Heat-clearing and detoxifying; promote diuresis; treat strangury	KL-153
111	*Hydrangea macrophylla* (Thunb.) Ser	Yuan zhi xiu qiu	ghab lenl hab	Saxifragaceae	Leaf, root	Grinding, decoction	Detoxification; hemostasis	KL-103
112	*Hypericum patulum* Thunb. ex Murray	Jin si mei	vob nix ngol	Guttiferae	Whole plant	Oral	Heat-clearing and detoxifying; activating blood circulation to dissipate; eliminating phlegm and stopping cough; blood cooling and arresting	KL-230
113	*Hypericum perforatum* L	Guan ye lian qiao	det bangx fangx	Guttiferae	Whole plant	Oral, grinding, decoction	Stop bleeding; heat-clearing and detoxifying; regulate the menstrual function; regulating menstruation; lactagogue	KL-157
114	*Impatiens crassiloba* Hook. f	Feng xian hua	bangx qangb	Balsaminaceae	Stems, root, flower	Oral, boiled with water, medicinal liquor	Dispelling wind and eliminating dampness; activating blood circulation and stimulating meridians; set a fracture	KL-060
115	*Impatiens crassiloba* Hook. f	Hou lie feng xian hua	bangx gent bil dab	Balsaminaceae	Flower	Oral, medicinal liquor	Clear the blood and the swelling away; analgesia	KL-152
116	*Imperata cylindrica* (L.) Beauv	Bai mao	nangx ghab lix	Gramineae	Tuber	Oral	Blood cooling and arresting; engender liquid and heat-clearing; promote diuresis; treat strangury	KL-102
117	*Inula helianthus-aquatica* C. Y. Wu ex Ling	Shui zhao yang	bangx mais hnaib	Compositae	Flower	Oral, boiled with meat and drunk the soup	Resolve phlegm; dispelling wind and eliminating dampness	KL-189
118	*Ixeris polycephala* Cass	Ku mai cai	vob ib	Compositae	Whole plant	Oral and external, grinding, decoction, medicated bath	Heat-clearing and detoxifying; decreasing swelling to relieving pain	KL-080
119	*Juncus effusus* L	Deng xin cao	nangx songb mil	Juncaceae	Stem, dried	Oral	Dituesis; treating stranguria	KL-229
120	*Kadsura longipedunculata* Finet et Gagnep	Nan wu wei zi	ghab jongx zeng ghongd yut	Schisandraceae	Fruit	Boiled with vinegar	Promoting blood circulation to remove meridian obstruction; decreasing swelling to relieving pain;	KL-158
121	*Kyllinga brevifolia* Rottb	Shui wu gong	nangx hsob nail	Cyperaceae	Whole plant	Oral and external, pound fresh part applied on the affected area, boiled with water	Preventing further attack of malaria; capable of preventing phlegm from forming and stopping coughing; expelling wind	KL-035
122	*Lagenaria siceraria* (Molina) Standl. var. *depressa* (Ser.) Hara	Hu lu	fab xef	Cucurbitaceae	Fruit	Oral, grinding, decoction	Treating stranguria and resolving mass; inducing diuresis for removing edema	KL-128
123	*Lasiosphaera fenxlii* Reich	Tuo pi ma bo	jib penb	Lycoperdaceae	Sporophore	Pound fresh part applied on the affected area	Clearing lung; detoxification; hemostatic	KL-219
124	*Lemna mino*r L	Fu ping	box niel	Lemnaceae	Whole plant	Taken orally soup	Relieving exterior syndrome by diaphoresis; promoting eruption and anti-pruritus; inducing diuresis for removing edema	KL-101
125	*Leonurus japonicus* Thunb	Yi mu cao	jab lob ghel hlieb	Labiatae	Whole plant	Oral, soup	Promoting blood circulation for regulating menstruation; diuresis detumescence	KL-151
126	*Ligustrum robustum* (Roxb.) Blume	Cu zhuang nv zhen	jenl ib	Oleaceae	Leaf	Taken orally soup; pound fresh part applied on the affected area	Clear liver fire; antipyretic drugs	KL-190
127	*Lilium brownii* F. E. Brown ex Miellez var. *viridulum* Baker	Bai he	bod gab tid	Liliaceae	Bulb	Grinding, decoction	Nourishing Yin and moistening lung; tranquilization	KL-036
128	*Litsea cubeba* (Lour.) Pers	Shan ji jiao	zend jangl	Lauraceae	Fruit, leaf, root, stem	Taken orally soup	Promoting flow of qi and blood circulation; anti-asthmatic; diuretic	KL-037
129	*Lobelia chinensis* Lour	Ban bian lian	uab berx nex nas	Campanulaceae	Whole plant	Oral, grinding and drink with wine	Heat-clearing and detoxifying; inducing diuresis for removing edema	KL-131
130	*Lonicera japonica* Thunb	Ren dong	bangx jab hxangd	Caprifoliaceae	Flower, rattan	Oral and external, medicated bath	Heat-clearing and detoxifying	KL-061
131	*Lophatherum gracile* Brongn	Dan zhu ye	niangx ghab nex gix	Gramineae	Whole plant	Oral, boiled with meat and drunk the soup	Heat-clearing and detoxifying; diuretic	KL-127
132	*Luffa cylindrica* (L.) Roem	Si gua	fab hsab	Cucurbitaceae	Fruit	Oral and external, pound fresh part applied on the affected area, boiled with water	Removing heat-phlegm; cooling blood remove pathogenic heat	KL-082
133	*Lycium chinense* Miller	Gou qi	det uab bol	Solanaceae	Fruit	Grinding, decoction	Nourishing liver and kidney; relieving dryness and moistening	KL-132
134	*Lycopodium japonicum* Thunb. ex Murray	Shi song	hsob git nail nib	Lycopodiaceae	Whole plant	Medicinal liquor; pound fresh part applied on the affected area	Expelling wind; relax the muscles and stimulate the blood circulation	KL-081
135	*Lycoris radiate* (L’Her.) Herb	Shi suan	ghax vib	Amaryllidaceae	Bulb	Oral, grinding, decoction, boiled with meat and drunk the soup	Antitussive; detoxification and detumescence	KL-100
136	*Lygodium japonicum* (Thunb.) Sw	Hai jin sha	jab hxangd	Lygodiaceae	Spore	Grinding, decoction	Dituesis; anti-febrile	KL-039
137	*Lysimachia christinae* Hance	Guo lu huang	vob nix ngol	Primulaceae	Whole plant	Grinding, decoction; pound fresh part applied on the affected area	Dituesis; treating stranguria; heat-clearing and detoxifying; removing stasis and detumescence	KL-040
138	*Lysimachia clethroides* Duby	Zhan zhu cai	jub maix vud	Primulaceae	Whole plant	Grinding and drink with wine; pound fresh part applied on the affected area	Clearing heat and promoting diuresis; active blood and disperse stagnation; inducing diuresis for removing edema; regulate the menstrual function; regulating menstruation	KL-099
139	*Lysimachia paridiformis* Franch. var. *stenophylla* Franch	Xiao ye luo di mei	kod tud vud	Primulaceae	Whole plant	Grinding, decoction	Promoting blood circulation to remove meridian obstruction; expelling wind to relive pain	KL-159
140	*Macleaya cordata* (Willd.) R. Br	Bo luo hui	vob liangl bab	Papaveraceae	Whole plant	Grinding, decoction	Promoting blood circulation to remove blood stasis; heat-clearing and detoxifying; insecticidal; anti-itch	KL-083
141	*Mahonia bealei* (Fort.) Carr	Kuo ye shi da gong lao	det hmib nangl	Berberidaceae	Leaf, root	Oral, boiled with water	Heat-clearing and detoxifying; dryness-heat	KL-115
142	*Melia toosendan* Sieb. et Zucc	Chuan lian	det zend ib	Meliaceae	Fruit	Taken orally soup; pound fresh part applied on the affected area	Insecticide	KL-116
143	*Mirabilis jalapa* L	Zi mo li	nuf suix fenx	Nyctaginaceae	Root	Grinding, decoction	Heat-clearing and detoxifying; promoting blood circulation	KL-191
144	*Nandina domestica* Thunb	Nan tian zhu	ghaob hold ghunb	Berberidaceae	Root, stem, Fruit	Oral, grinding, decoction	Clear away heat and remove dampness; clearing and activating the channels and collaterals; Cough and asthma	KL-084
145	*Oenanthe javanica* (Bl.) DC	Shui qin	vob juex	Umbelliferae	Whole plant	Taken orally soup; pound fresh part applied on the affected area	Heat-clearing and detoxifying; diuretic and hemostasis	KL-041
146	*Ophioglossum pedunculosum* Desv	Yi zhi jian	wab kaob naob	Ophioglossaceae	Whole plant	Grinding, decoction	Hemorrhoids; venomous snake bite; traumatic injury; infantile malnutrition	KL-126
147	*Ophiopogon japonicus* (L. f.) Ker-Gawl	Mai dong	zend jab ngol yut	Liliaceae	Tuber	Grinding, decoction	Tonifying stomach and promoting fluid; nourishing Yin and moistening lung	KL-062
148	*Opuntia stricta* (Haw.) Haw. var. dillenii (Ker-Gawl.) Benson	Xian ren zhang	ghab jongx vob nix	Cactaceae	Stem	Oral, grinding and drink with wine	Promoting flow of qi and blood circulation; heat-clearing and detoxifying	KL-044
149	*Origanum vulgare* L	Niu zhi	reib nzeal youl	Labiatae	Whole plant	Oral, soup	Clearing summer-heat; inducing diuresis for removing edema	KL-085
150	*Osbeckia opipara* C. Y. Wu et C. Chen	Chao tian guan	jab tok	Melastomataceae	Root	Taken orally soup	The blood circulation hematischesis	KL-098
151	*Osmunda japonica* Thunb	Zi qi	vob haid ghab dliangb	Osmundaceae	Root, stems, leaf	Grinding, decoction	Heat-clearing and detoxifying; blood cooling and arresting; insecticide	KL-205
152	*Paris polyphylla* Smith	Qi ye yi zhi hua	jab gib liod	Liliaceae	Tuber	Boiled with water; pound fresh part applied on the affected area	Clearing heat and detoxicating; dispelling wind and relieving convulsion	KL-192
153	*Patrinia scabiosaefolia* Fisch. ex Trev	Bai jiang	jab zangd naib	Valerianaceae	Whole plant	Grinding and decoction the young leaves	Heat-clearing and detoxifying; apocenosis; promoting blood circulation	KL-228
154	*Perilla frutescens* (L.) Britt	Zi su	ghab vud	Labiatae	Whole	Oral	Relieving exterior syndrome; remove coldness; regulating the flow of qi to alleviate pain	KL-227
155	*Periploca forrestii* Schltr	Xi nan gang liu	ghab bas hlat dlaib	Gramineae	Whole plant	Oral and external, pound fresh part applied on the affected area, boiled with water	Relax the muscles and stimulate the blood circulation; dispelling wind and eliminating dampness	KL-02
156	*Peristrophe japonica* (Thunb.) Bremek	Jiu tou shi zi cao	nangx zend naf	Acanthaceae	Whole plant	External, medicinal liquor or medicated bath	Wind–dampness dispelling and detoxification	KL-206
157	*Pharbitis purpurea* (L.) Voigt	Yuan ye qian niu	vob hmuk vongx	Convolvulaceae	Seed	Oral, grinding, decoction	Dituesis and purgation; make expectoration easy; disperse accumulations; insecticide	KL-097
158	*Phytolacca americana* L	Chui xu shang lu	vob bix gheib	Phytolaccaceae	Root	Grinding, decoction	Restoring vital energy; dituesis	KL-148
159	*Pinellia ternata* (Thunb.) Breit	Ban xia	kod las	Araceae	Tuber	Oral and external, pound fresh part applied on the affected area, boiled with water	Eliminating dampness and reducing phlegm; calm the adverse-rising energy; check retching; relieving and eliminating mass	KL-043
160	*Plantago asiatica* L	Che qian	vob naix bat dliangt	Plantaginaceae	Seed	Taken orally soup; pound fresh part applied on the affected area	Diuretic; relieving exterior and promoting dampness; removing liver fire for improving eyesight; cooling blood remove pathogenic heat	KL-193
161	*Platycarya strobilacea* Sieb. et Zucc	Hua xiang shu	det jab jib	Juglandaceae	leaf	External, grinding and drink with wine	Detoxification; insecticidal; anti-itch	KL-149
162	*Platycladus orientalis* (L.) Franco	Ce bai	det hxangb	Cupressaceae	Leaf, fruit	Oral, grinding, decoction	Blood cooling and arresting; resolve phlegm to relive cough; expelling wind–damp	KL-150
163	*Polygala japonica* Houtt	Gua zi jin	vob nil lios bad	Polygalaceae	Whole plant	Taken orally soup; pound fresh part applied on the affected area	Resolve phlegm to relive cough; promoting blood circulation; detumescence; tranquilization; detoxification	KL-147
164	*Polygonatum cyrtonema* Hua	Duo hua huang jing	kid vud	Liliaceae	Tuber	Grinding, decoction	Nourishing Yin and moistening lung; invigorating spleen and replenishing qi	KL-045
165	*Polygonum aviculare* L	Bian xu	vob jab ghab qangf	Polygonaceae	Whole plant	Grinding, decoction	Dituesis; treating stranguria	KL-207
166	*Polygonum capitatum* Buch.-Ham. ex D. Don	Tou hua liao	dlob dongd xok	Polygonaceae	Whole plant	Medicated bath	Heat-clearing and detoxifying; promote diuresis; treat strangury; promoting blood circulation and stopping pain	KL-208
167	*Polygonum cuspidatum* Sieb.et Zucc	Hu zhang	vob gongx liongl	Polygonaceae	Tuber, root	Grinding, decoction	Promoting blood circulation to remove blood stasis; heat-clearing and detoxifying; dispelling wind and eliminating dampness;	KL-146
168	*Polygonum hydropiper* L	Shui liao	vob liof	Polygonaceae	Whole plant	Taken orally soup; medicated bath	Detoxification; clearing damp; hemostasis	KL-194
169	*Polygonum perfoliatum* L	Kang ban gui	jab eb wal nangl	Polygonaceae	Aerial part, dried	Taken orally soup	Heat-clearing and detoxifying; dissolving stasis and hemostasis	KL-160
170	*Portulaca oleracea* L	Ma chi xian	vob hmid nangx	Portulacaceae	Aerial parts	Pound fresh part applied on the affected area	Heat-clearing and detoxifying; cool the blood; check dysentery; xeransis	KL-226
171	*Potentilla chinensis* Ser	Wei ling cai	vob hob dlub	Rosaceae	Whole plant	Grinding, decoction; pound fresh part applied on the affected area	Cool the blood and check dysentery; heat-clearing and detoxifying	KL-117
172	*Potentilla kleiniana* Wight et Arn	She han wei ling cai	jab eb wal nangb	Rosaceae	Whole plant	Taken orally soup; medicated bath	Heat-clearing and detoxifying; relieves cough and reduced phlegm herb; decreasing swelling to relieving pain; preventing further attack of malaria	KL-225
173	*Pratia nummularia* (Lam.) A. Br. et Aschers	Tong chui yu dai cao	zid hmangb lab	Campanulaceae	Whole plant	Oral and external, grinding and drink with wine, medicated bath	Dispelling wind and eliminating dampness; detoxification	KL-161
174	*Prunella vulgaris* L	Xia ku cao	ried dend longx	Labiatae	Ear, dried	Oral	To produce an effect toward clear vision; removing swelling and lump	KL-046
175	*Pteris cretica* L. var. *nervossa* (Thunb.) Ching et S.H.Wu	Feng wei jue	vob haib ghab mox	Pteridaceae	Whole plant	Grinding, decoction	Clearing heat and promoting diuresis; blood cooling and arresting; removing toxicity for detumescence	KL-209
176	*Pueraria lobate* (Willd.) Ohwi	Ye ge	ghab jongx hfib	Leguminosae	Tuber	Grinding, decoction	Anti-febrile; relieving exterior syndrome; promoting eruption and promoting spleen yang	KL-145
177	*Pyracantha fortuneana* (Maxim.) Li	Huo ji	zend gangb kongb	Rosaceae	Fruit, root, leaf	Grinding, decoction	Strengthening spleen; improve digestion; analgesia; check dysentery	KL-162
178	*Pyrrosia sheareri* (Baker) Ching	Lu shan shi wei	vob nix liod	Polypodiaceae	Whole plant	Taken orally soup	Blood cooling and arresting; clearing lung and eliminating phlegm; dituesis; treating stranguria	KL-063
179	*Rabdosia lophanthoides* (Buch.-Ham. ex D. Don) Hara	Xian wen xiang cha cai	gad hniangd vud	Labiatae	Whole plant	Oral, soup	Clearing heat and promoting diuresis; cooling blood and removing stasis; insecticide	KL-210
180	*Rabdosia rubescens* (Hemsl.) Hara	Sui mi ya	nangx bait pet	Labiatae	Whole plant	Oral and external, pound fresh part applied on the affected area, boiled with water	Heat-clearing and detoxifying; promoting blood circulation and stopping pain	KL-096
181	*Ranunculus japonicus* Thunb	Mao gen	jab mongb hfud seil	Ranunculaceae	Whole plant	Boiled with water; pound fresh part applied on the affected area	Jaundice; postoperative analgesia	KL-163
182	*Rhus chinensis* Mill	Yan fu mu	zend ghob pad	Anacardiaceae	Tuber	Oral and external medicated bath, grinding, decoction	Dispelling wind and eliminating dampness; inducing diuresis for removing edema; the blood circulation hematischesis; detoxification; antitussive	KL-143
183	*Ricinus communis* L	Bi ma	zend gangb hseik liod	Euphorbiaceae	Seed	Oral, boiled with water	Detumescence; activating collateral	KL-237
184	*Rohdea japonica* (Thunb.) Roth	Wan nian qing	uab fangf	Liliaceae	Root	Taken orally soup; grinding and drink with wine; pound fresh part applied on the affected area	Heat-clearing and detoxifying; relieve pain; stasis	KL-144
185	*Rosa chinensis* Jacq	Yue ji	bangx bei liangx	Rosaceae	Flower	Taken orally soup	Promoting blood circulation for regulating menstruation; removing toxicity for detumescence; enhancing splenic function; hemostasis	KL-224
186	*Rosa cymosa* Tratt	Xiao guo qiang wei	qangf weif zend yut	Rosaceae	Root, fruit, leaf	Grinding, decoction	Arnica Extract; drainage and detoxification	KL-223
187	*Rosa laevigata* Michx	Jin ying zi	bel liangx	Rosaceae	Root, fruit	Grinding, decoction; medicated bath	Secure essence; astringe the intestines; check vaginal discharge	KL-095
188	*Rosa roxburghii* Tratt	Sao si hua	ghab jongx det bel tok	Rosaceae	Root	Grinding, decoction	Strengthening spleen; good for aiding digestion; antitussive; anti-diarrhea effect	KL-164
189	*Rostellularia procumbens* (L.) Nees	Jue chuang	det nix nied	Acanthaceae	Whole plant	Boiled with hot water	Heat-clearing and detoxifying; promoting blood circulation and urination with diuretics; disperse accumulations; analgesia	KL-119
190	*Rubia lanceolata* Hayata	Pi zhen ye qian cao	vob niangx hxib	Rubiaceae	Root	Grinding, decoction	Blood cooling and arresting; active blood and disperse stagnation	KL-211
191	*Rubus corchorifolius* L. f	Shan mei	zend liul vob	Rosaceae	Root, leaf	Boiled with hot water	Hemostasis; check vaginal discharge; check vaginal discharge; anti-pruritus	KL-118
192	*Rubus setchuenensis* Bureau et Franch	Chuan mei	zend lil	Rosaceae	Root	Medicinal liquor; boiled with water; boiled with meat and drunk the soup	Dispelling wind and eliminating dampness; cool the blood; the blood circulation hematischesis; produce the muscle and heal ulcer	KL-094
193	*Rumex nepalensis* Spreng	Ni bo er suan mo	vob haib hxub	Polygonaceae	Root, leaf	Medicated bath	Heat-clearing and detoxifying; cool the blood; insecticide; purgation	KL-140
194	*Sabia parviflora* Wall. ex Roxb	Xiao hua qing feng teng	hlat det lod ninx	Sabiaceae	Stem, leaf	Taken orally soup; pound fresh part applied on the affected area	Anti-inflammatory and analgesia; clearing heat and promoting diuresis; clearing liver to add yin; expelling wind–damp; cholagogue	KL-047
195	*Sagina japonica* (Sw.) Ohwi	Qi gu cao	jangx lul vongx	Caryophyllaceae	Whole plant	Oral, boiled with water	Cooling blood remove pathogenic heat; reducing swelling and resolving mass; insecticidal	KL-120
196	*Salvia splendens* Ker-Gawl	Yi chuan hong	ib zongs xok	Labiatae	Whole plant	Oral, soup	Removing toxicity for detumescence; cool blood and nourish yin	KL-093
197	*Salvia yunnanensis* C. H. Wright	Yun nan shu wei cao	hxangt gheib	Labiatae	Root	Oral, boiled with meat and drunk the soup	Removing stasis and promoting tissue regeneration; blood cooling and arresting; promoting blood circulation for regulating menstruation; detumescence	KL-092
198	*Sanguisorba officinalis* L	Di yu	vob ot wel	Rosaceae	Root	Grinding and drink with wine	Blood cooling and arresting; heat-clearing and detoxifying; heal ulcer; detumescence	KL-165
199	*Sarcandra glabra* (Thunb.) Nakai	Cao shan hu	det nix vub hlieb	Chloranthaceae	Whole plant	Oral and external, pound fresh part applied on the affected area, boiled with water	Promoting blood circulation to remove blood stasis; anti-febrile; set a fracture	KL-121
200	*Sargentodoxa cuneata* (Oliv.) Rehd. et Wils	Da xue teng	hsob hxangt	Sargentodoxaceae	Root	Grinding, decoction; boiled with water	Dispelling wind and eliminating dampness; promoting blood circulation and stopping pain; insecticide; detoxification	KL-166
201	*Saxifraga stolonifera* Curt	Hu er cao	vob bix seix	Saxifragaceae	Whole plant	Pound fresh part applied on the affected area	Can be wind-dispersing heat; cooling blood remove pathogenic heat	KL-212
202	*Selaginella uncinata* (Desv.) Spring	Cui yun cao	jab cangt jent	Selaginellaceae	Whole plant	Boiled with water; medicated bath	Clearing heat and promoting diuresis; detoxification; hemostasis	KL-091
203	*Semiaquilegia adoxoides* (DC.) Makino	Tian kui	jab ghad nangl	Ranunculaceae	Tuber	Grinding, decoction	Heat-clearing and detoxifying; promoting blood circulation to remove blood stasis; phlegm-and mass-eliminating; dituesis	KL-215
204	Senecio scandens Buch.-Ham. ex D. Don	Qian li guang	vob wik nax	Compositae	Whole plant	Oral and external, grinding, decoction, medicated bath	Clearing heat and detoxicating; expelling blood stasis for improving eyesight; expelling blood stasis for improving eyesight	KL-167
205	*Serissa serissoides* (DC.) Druce	Bai ma gu	det vil gheib	Rubiaceae	Whole plant	Grinding, decoction	Dispelling wind and eliminating dampness; heat-clearing and detoxifying;	KL-048
206	*Siegesbeckia pubescens* Makino	Xian geng xi xian	vob bix hnaib	Compositae	Whole plant	Oral and external, grinding and drink with wine	Expelling wind–damp; relaxing tendon and activation collaterals; heat-clearing and detoxifying	KL-064
207	*Sinosenecio oldhamianus* (Maxim.) B. Nord	Pu er gen	ghab jongx puf eef	Compositae	Whole plant	Oral and external, grinding and drink with wine	Detoxification; promoting blood circulation	KL-065
208	*Smilax china* L	Ba qia	vob dlod dlof	Liliaceae	Leaf	Grinding, decoction	Dispelling wind and eliminating dampness; diuretic; promoting blood circulation to remove blood stasis; detoxification	KL-090
209	*Smilax glabra* Roxb	Tu fu ling	bod zangd dak	Liliaceae	Tuber, root	Grinding, decoction	Dispel dampness and resolve toxin	KL-222
210	*Solanum lyratum* Thunb	Bai ying	jab diel vud niab	Solanaceae	Whole plant	Boiled with meat and drunk the soup	Expelling wind; detoxification	KL-214
211	*Sophora flavescens* Alt	Ku shen	jab gongx saib	Leguminosae	Root	Grinding, decoction	Heat-clearing and damp-drying drug; insecticide; diuretic	KL-213
212	*Spiraea japonica* L. f	Fen hua xiu xian ju	vob sob diel	Rosaceae	Root	Grinding, decoction	Expelling wind and clearing heat; improving eyesight and removing nebula	KL-066
213	*Spiranthes sinensis* (Pers.) Ames	Shou cao	ghab jongb linl hlob hlaob	Orchidaceae	Root, whole plant	Grinding, decoction	Nourishing yin and cooling blood; moistening lung for arresting cough; enhancing qi while nourishing fluid	KL-030
214	*Stemona tuberosa* Lour	Dui ye bai bu	vob ghab dail lix	Stemonaceae	Tuber	Medicinal liquor	Moistening lung for arresting cough; insecticide	KL-123
215	*Stenoloma chusanum* Ching	Wu jue	det mangs hsang	Lindsaeaceae	Whole plant	Grinding, decoction	Heat-clearing and detoxifying; removing dampness and arresting bleeding	KL-122
216	*Stephania cepharantha* Hayata	Jin xian diao wu gui	jab fangx liangx	Menispermaceae	Tuber	Taken orally soup; grinding and drink with wine; pound fresh part applied on the affected area	Heat-clearing and detoxifying; expelling wind and stopping pain; blood cooling and arresting	KL-029
217	*Talinum paniculatum* (Jacq.) Gaertn	Tu ren shen	vob eb bens	Portulacaceae	Root	Taken orally soup	Invigorate the spleen and promoting blood; menstrual extraction; moistening lung for arresting cough	KL-067
218	*Taraxacum mongolicum Hand.-Mazz*	Pu gong ying	uab berx ferx	Compositae	whole plant	Oral, boiled with water	Heat-clearing and detoxifying; detumescence and Stasis	KL-070
219	*Tetrapanax papyrifer (Hook.) K. Koch*	Tong tuo mu	det bel tingd	Araliaceae	Stem, root	Oral, boiled with water	Clearing heat; dituesis; lactagogue	KL216
220	*Tinospora sagittata*(Oliv.)Gagnep	Qing niu dan	bad jex sangx	Menispermaceae	Tuber	Grinding, decoction; grinding and drink with wine; pound fresh part applied on the affected area	Heat-clearing and detoxifying; decreasing swelling to relieving pain	KL-089
221	*Toddalia asiatica* (L.)Lam	Fei long zhang xue	ghab jongx bel sob xok gax bas	Rutaceae	Root	Grinding and drink with wine; taken orally soup; pound fresh part applied on the affected area	Arnica extract; dispelling wind and eliminating dampness; set a fracture; analgesia	KL-028
222	*Toona sinensis* (A. Juss.) Roem	Xiang chun	vob yangl	Meliaceae	Bark	Grinding, decoction	Heat-clearing and damp-drying drug; astringe the intestines; hemostasis; check vaginal discharge; insecticide	KL-088
223	*Toricellia angulata* Oliv. var. *intermedia* (Harms.) Hu	You chi qiao bing mu	ghab jongx linl det diol	Cornaceae	Root, bark, leaf	Oral, grinding, decoction	Promoting blood flow and tendon relaxation; dispelling wind and eliminating dampness	KL-218
224	*Trachycarpus fortunei* (Hook.) H. Wendl	Zong lü	det hsob	Palmae	Fruit, leaf	Grinding, decoction	Antitussive; hemostasis	KL-124
225	*Trichosanthes kirilowii* Maxim	Gua lou	zend fab hvub	Cucurbitaceae	Root, fruit	Oral, grinding, decoction	Removing heat-phlegm; relieving dryness with moistening drugs; loosen the chest and dissipate binds	KL-217
226	*Tripterospermum cordatum* (Marq.) H. Smith	Xin ye shuang hu die	jab juf saix	Gentianaceae	Whole plant	External, grinding, drink with wine	Invigorating spleen and clearing away heat to relieving wet; insecticide; wind-heat	KL-125
227	*Typha angustifolia* L	Shui zhu	nangx laf zuf	Typhaceae	Pollen	Grinding, decoction; pound fresh part applied on the affected area	Diuretic and hemostasis; removing stasis	KL-068
228	*Valeriana jatamansi* Jones	Zhi zhu xiang	vob gangb vas	Valerianaceae	Stem, root, dried	Grinding, decoction	Manage qi and activating blood; detumescence; dehumidification	KL-221
229	*Verbena officinalis* L	Ma bian cao	jab lob gheib	Verbenaceae	Whole plant	Grinding, decoction; taken orally soup; medicated bath	Heat-clearing and detoxifying; promoting blood circulation and stopping pain; inducing diuresis for removing edema	KL-065
230	*Vernicia fordii* (Hemsl.) Airy-Shaw	You tong	bangx zend yux	Euphorbiaceae	Root, leaf, flower	Oral and external, pound fresh part applied on the affected area, boiled with water	Detumescence; removing stasis; insecticide	KL-141
231	*Veronica didyma* Tenore	Po po na	nangx vux denb	Scrophulariaceae	Whole plant	Taken orally soup	Tonifying kidney; strengthen waist and sinews; removing toxicity for detumescence	KL-027
232	*Vicia cracca* L	Guang bu ye wan dou	def xux vud	Leguminosae	Whole plant	Boiled with water; pound fresh part applied on the affected area	Dispelling wind and eliminating dampness	KL-219
233	*Vitex negundo* L	Huang jing	ndut ghunx leb	Verbenaceae	Root	Grinding, decoction	Heat-clearing and detoxifying	KL-087
234	*Xanthium sibiricu*m Patrin ex Widder	Cang er	jab vub	Compositae	Fruit, stem, leaf	Oral, grinding, decoction, boiled with meat and drunk the soup	Dispelling wind and eliminating dampness; digestion; analgesia	KL-069
235	*Zanthoxylum armatum* DC. var. *ferrugineum* (Rehd.et Wils.) Huang	Zhu ye jiao	ghab jongx zend sob vud	Rutaceae	Fruit	Grinding, decoction	Relieving rheumatism and cold; invigorating blood circulation and stopping pains; antitussive	KL-142
236	*Zanthoxylum bungeanum* Maxim	Hua jiao	zend sob	Rutaceae	Peel	Taken orally soup; medicated bath	Insecticidal; anti-diarrhea effect; eliminating dampness	KL-220
237	*Zingiber officinale* Roscoe	Jiang	kid	Zingiberaceae	Tuber	Grinding, decoction; taken orally soup; boiled with meat and drunk the soup086	Dispelling cold; calm the adverse-rising energy; check retching; eliminating phlegm and stopping cough	KL-026

### Diseases treated by products from traditional markets

The marketplace and source locations of medicinal plants are in southwest China, with high humidity, moderate temperature, varied terrain, and abundant wild plant resources. The medicinal plants traded on the market were used to treat 83 human ailments. Traumatic injuries have been treated with 73 species of medicinal plants, followed by skin diseases (40 species), cough (36 species), rheumatism (34 species), digestion (25 species), and gynecological conditions (23 species). A large number of medicinal plants (96 species) are used for heat-clearing, a TCM disease category, and detoxifying.

All the local therapeutic uses of medicinal plants were grouped into 20 medical categories, and a FIC value was computed for each (Table 5). The FIC values ranged between 0.36 and 0.95 demonstrating high levels of consensus among the 116 vendors for multiple uses of 237 medicinal plant species sold. The inflammation category had the highest FIC value of 0.95, showing a high level of agreement among the 116 vendors for the 5 medicinal plant species sold to treat inflammation. This indicated that these plants were well known by the vendors, suggesting that they may have a significant effect on treating inflammatory diseases. Other diseases also had high FIC values, including treatments for stomach, intestine, and liver diseases (0.82), heart and circulatory system diseases (0.81), and fever and malaria (0.80). The lowest FIC values recorded in this study included treatments for respiratory diseases (0.36) and cough (0.36).

**Table 5:** Informant consensus factor by categories of diseases in the study area

Categories of diseases	Number of medicinal plant species	Percentage of all medicinal plant species (%)	Use citations by market vendor	Percentage of all use citations (%)	FIC
Inflammation	5	0.87	83	5.67	0.95
Stomach, intestine, and liver diseases (Internal Organ)	12	2.08	61	4.16	0.82
Heart and circulatory system	4	0.69	17	1.16	0.81
Fever and malaria	3	0.52	11	0.75	0.8
Pain	17	2.95	78	5.32	0.79
Traumatic injury and sprain	73	12.67	298	20.34	0.76
Heat-clearing and detoxifying	96	16.67	307	20.96	0.69
Rheumatic problems	34	5.90	101	6.89	0.67
Male problems	5	0.87	13	0.89	0.67
Skin diseases, skin cut, and wound	40	6.94	87	5.94	0.55
Dysentery	12	2.08	25	1.71	0.54
Ocular disease	7	1.22	14	0.96	0.54
Digestion	25	4.34	52	3.55	0.53
Gynecological problems	23	3.99	43	2.94	0.48
Cough	36	6.25	56	3.82	0.36
Respiratory system	17	2.95	26	1.77	0.36
Other uses	12	2.08	18	1.23	0.35

**Fig. 5:**
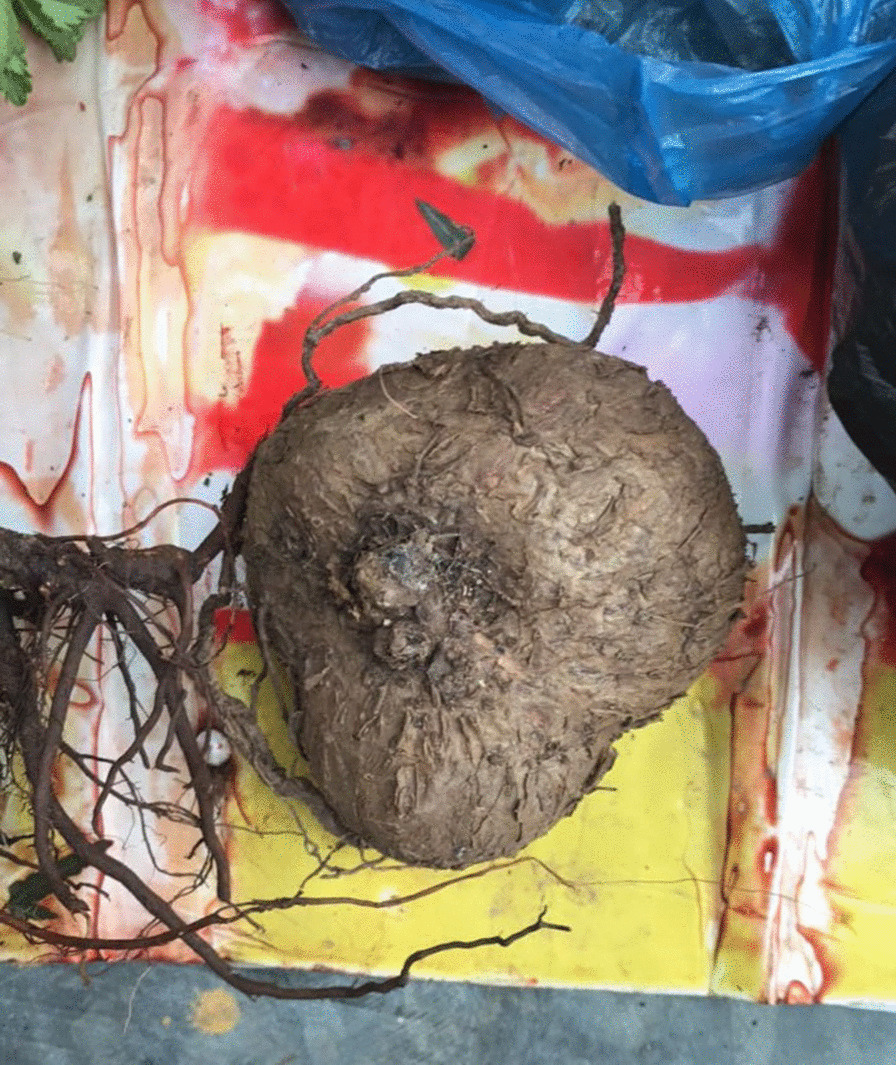
Root of *Stephania cepharantha* sold at the market. Photo by S Liu

**Fig. 6:**
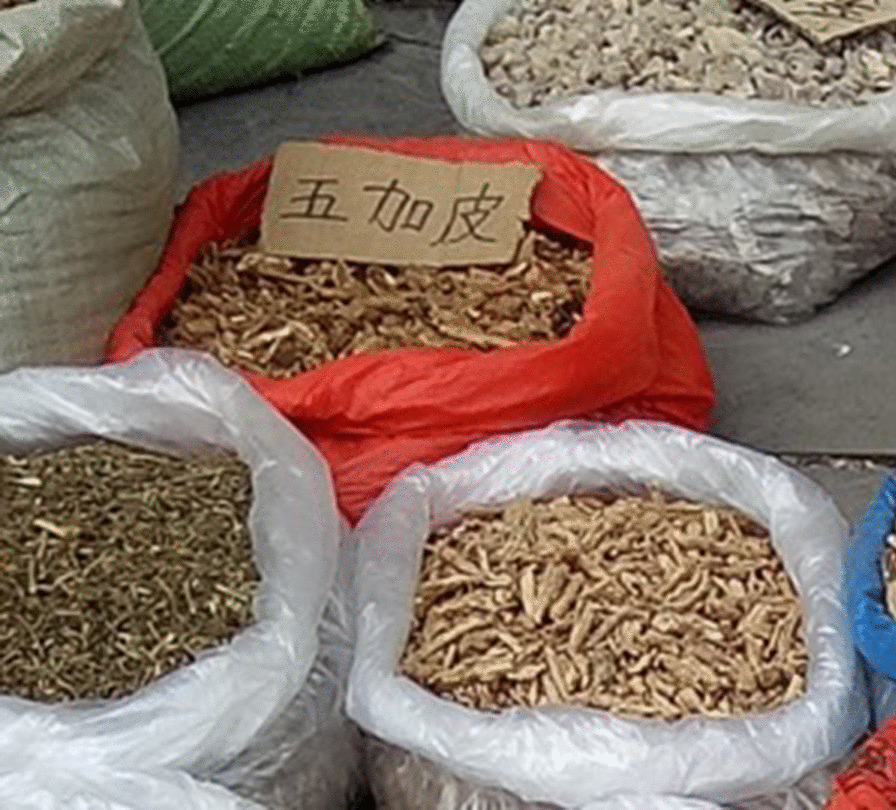
Slices of *Eleutherococcus nodiflorus* sold at the market. Photo by S Liu

**Fig. 7:**
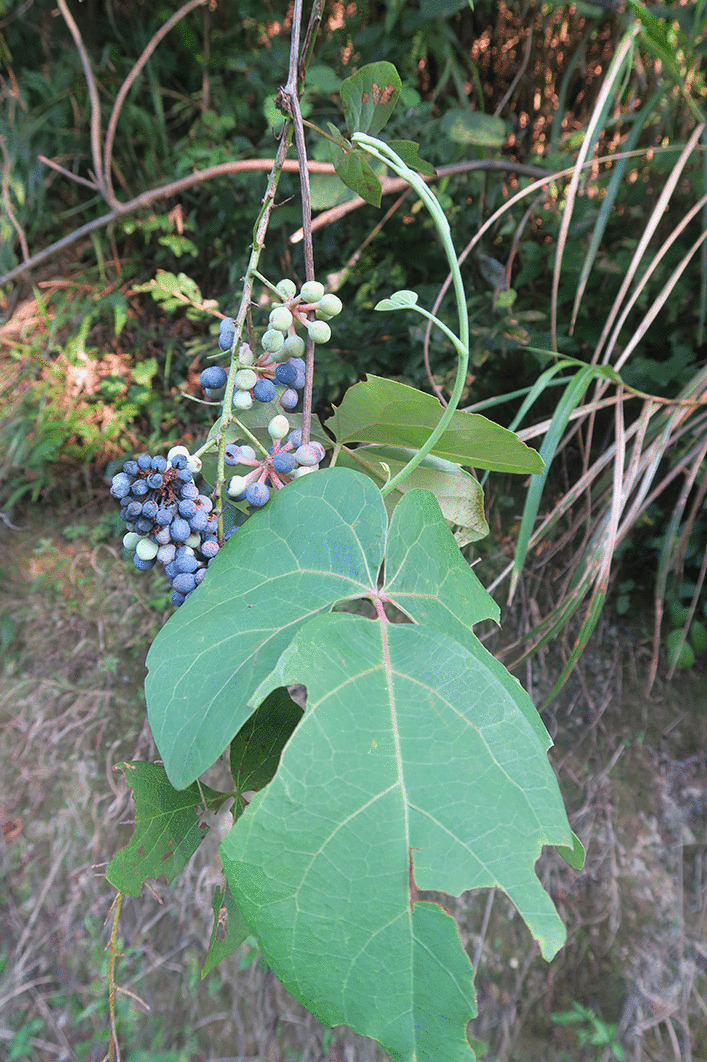
Living plant of *Sargentodoxa cuneata*. Photo by C Long

The FL index indicates that there are 15 important medicinal plant species (Table 6) in the Kaili market, according to the information provided by 20 market suppliers for the treatment of 20 diseases. In this analysis, 237 species of medicinal plants mentioned by vendors were calculated. Three medicinal plant species with FL > 90% include *Stephania cepharantha* (Fig. 5), *Eleutherococcus nodiflorus* (Fig. 6), and *Sargentodoxa cuneata* (Fig. 7) are used for conditions like sprains/traumas, rheumatism, and heat/detoxification. This high FL may be related to their success in the treatment of these diseases and/or to the local cultural practices. Nine medicinal plants, including the previous three, had an FL > 70%. The additional species were *Fallopia multiflora*, *Gleditsia sinensis, Grangea maderaspatana*, *Polygonum perfoliatum*, *Saxifraga stolonifera*, and *Stenoloma chusanum*.

**Table 6:** Most used medicinal plant species for medical categories based on the highest fidelity level from Kaili market

No.	Medicinal plant species	Medical category	Ip	Iu	FL value (%)
1	*Eleutherococcus nodiflorus*	Traumatic injury and sprain	91	95	0.96
2	*Sargentodoxa cuneata*	Rheumatic problems	77	83	0.93
3	*Stephania cepharantha*	Heat-clearing and detoxifying	81	89	0.91
4	*Saxifraga stolonifera*	Heat-clearing and detoxifying	52	61	0.85
5	*Polygonum perfoliatum*	Skin diseases, skin cut, and wound	53	63	0.84
6	*Grangea maderaspatana*	Dysentery	31	37	0.84
7	*Fallopia multiflora*	Fever and malaria	11	15	0.73
8	*Gleditsia sinensis*	Cough	26	37	0.70
9	*Stenoloma chusanum*	Bleeding and hemorrhages	37	53	0.70
10	*Leonurus japonicus*	Gynecological problems	39	57	0.68
11	*Pyracantha fortuneana*	Dysentery	25	39	0.64
12	*Lycium chinense*	Stomach, intestine, and liver diseases (internal organ)	31	49	0.63
13	*Berberis julianae*	Inflammation	31	51	0.61
14	*Gynostemma pentaphyllum*	Inflammation	42	73	0.58
15	*Toddalia asiatica*	Pain	27	48	0.56

## Discussion

### Medicinal plants and associated traditional knowledge

Based on market surveys and field investigations, we documented 237 species (belonging to 219 genera and 107 families) of medicinal plants traded at Kaili traditional medicinal market. The number of medicinal plants sold indicates the rich indigenous knowledge of medicinal plants and their applications. The 145 herbaceous species form the biggest category of plant type. This may be because herbaceous plants are easier to collect and other forest resources are dwindling in abundance rapidly. This finding is similar to other studies in other areas [34–36]. Most vendors would use the whole plant for medicinal purposes, but such collection practices likely reduce the wild population. Like other linguistic groups, the Miao people also have the custom of collecting medicinal plants for cooking and bathing on the Dragon Boat Festival (the fifth day of the fifth month in the lunar calendar), including *Acorus calamus*, *Dipsacus asperoides*, *Paederia scandens,* and *Leonurus artemisia*. Many plants are harvested in the season around the Dragon Boat Festival [37], and thus it is the most prosperous time on Kaili medicinal market.

*Acorus calamus* is widely used by the Miao not only as a medicinal herb, but also it is used symbolically to ward off evil spirits by displaying it on doors or using it in a medicinal bath. The Miao healers often use the rhizomes of *Acorus calamus* to treat aphasia, traumas, diarrhea, snake bites, and stomach ache [38]. Some local Miao people soak their feet daily in hot water baths infused with *Acorus calamus* rhizomes to drive the cold away, balance *yin* and *yang*, and boost their immunity. When an elder dies, the Miao people boil *Acorus calamus* in water to scrub the corpse. They believe that *Acorus calamus* water will wash away unhappiness and allow the deceased to rest peacefully.

Some medicinal plant species are traditionally used as starters for preparing fermented beverages by Miao people, similar to a practice in the Shui communities [39]. In Kaili, the fifth and eighth months of the lunar calendar are considered the best times to buy wild fruits of *Ficus tikoua*, *Actinidia chinensis*, *Rubus setchuenensis*, and *Rosa roxburghii* for brewing wine or liquor.

Most of the knowledge on herbal remedies is handed down orally to the young people in the community by elders. In this market, 66.4% of vendors were male, probably because women are dedicating themselves to housework, childcare, keeping livestock, and farmyard management, while the men collect wild medicinal herbs from the high mountains [40]. Most medicinal plant vendors are small retailers who have common knowledge of Miao medicine and other ethnomedicine.

### Therapeutic effectiveness and popularity of medicinal plants

The medicinal plants from the market were used to treat 83 human ailments. Traumas, fevers, and skin diseases, for example, were common conditions among the Miao, which likely relates to their environment and culture [20]. Many Miao people face difficult living conditions in mountainous areas. When the Miao work in rugged mountainous terrain, they can be injured easily. That is likely the reason that herbs to treat traumatic injury occupy a large proportion of the medicinal market. The weather in Kaili and surrounding areas is wet and humid throughout the year [41]. From the theory of traditional Chinese medicine, those who live in damp areas should expel wind in the body regularly to relieve constipation and improve sleep quality, and then make their bodies feel better [42]. Thus, Miao people use many herbs to treat rheumatism. The Miao’s living environment is also regarded to cause so-called heat, another concept from TCM; thus, the medicinal plants for heat-clearing and detoxifying are very popular in the market.

Inflammation was cited as the highest number of medicinal plants, revealing the importance of anti-inflammatory treatment. In poor Miao villages, many people do not have the resources to purchase modern pharmaceuticals, so collection of herbs to treat inflammation is necessary. As a result, minor diseases can escalate to much more serious ones. Stomach, intestine, and liver diseases all have an FIC of = 0.82, showing a high level of agreement among the 116 vendors to treat these diseases.

The high FL values in this study highlight that the local vendors and residents have a strong dependence on these 15 species of medicinal plants. As such, all of these medicinal plants should be further studied, focusing on their chemistry, pharmacology, biological activity, and toxicity, as well as evaluation of efficacy and safety of local medicinal plants. For example, *Eleutherococcus nodiflorus*, *Sargentodoxa cuneata,* and *Stephania cepharantha* had an FL > 90%, which were used to treat traumatic injury and sprain, rheumatic problems, and heat-clearing and detoxifying. The most important nine species had an FL > 70%, have considerable agreement among market vendors on their particular use and credibility, and therefore could be further analyzed for potential development. Identifying plants with high values of FIC and FL is very important, as it will useful to support traditional medicine and establish related policies.

### Preparation and dosage of medicinal plants-based remedies

The Miao people use fresh medicinal plants frequently [43], while dry plants are seldom used. This is because they believe the active ingredients of fresh plants are still intact, so this method can optimize effectiveness [44]. The Miao healers usually mixed several species instead of a single herb. For example, a Miao healer may treat cold with *Dichondra repens*, *Arctium lappa*, *Taraxacum mongolicum*, and *Lonicera japonica*, instead of using a specific single plant species. Rheumatism and traumatic injury were the most common problems for which the Miao people prepare remedies with more than one plant species. When administering medicinal herbs, some healers practice a form of personalized medicine by preparing dosages according to individual patients, rather than measuring consistent doses.

The Miao usually use processing methods such as decoction, medicinal liquor, external application, and medicated bath. Medicinal plants are often added to food with an egg or animal meat for the purpose of enhancing the body’s immunity and supplement protein.

Meanwhile, the Miao people use different additives like alcohol, honey, salt, and sugar to improve the flavor and taste. In particular, the practice of combining plants and alcohol has a long history in Miao medicine. Miao healers use different procedures to administer their raw material/alcohol combinations. The medicinal plants are soaked in alcohol for about one month, and the resulting liquid then is drunk by the patient or applied externally to the affected parts. Alcohol can act as solvent instead of water, where fresh plant or dried plant powder is placed in alcohol and either drunk or applied externally [45]. It is believed that alcohol extracts contain more active components from the medicinal plants than water does, thus being more effective in curing diseases. For example, *Alangium chinense*, when soaked in alcohol, is far more effective in treating rheumatism than the fresh plant alone.

### Threats to medicinal plants and associated traditional knowledge

Compared with other herbal markets that only appear on the Dragon Boat Festival and Chung Yeung Festival (the 9^th^ day in September of lunar calendar), the Kaili medicinal market sold herbal medicines every week. These plants are in great demand and supply is limited. Lacking relevant development policies and protection measures in this area pose a serious problem, as some rare or endangered species were being sold. For example, *Paris polyphylla* is a common Miao medicinal plant in Guizhou. However, due to over-exploitation, the survival of wild populations is seriously threatened, and resources are dwindling. Therefore, the collection of plant resources and ex situ conservation of rare and endangered species are important missions, and selling endangered species in the market should be also controlled.

Most Miao medicinal knowledge was handed down orally to the younger members of the community by elders [46]. However, nowadays, indigenous knowledge is less commonly passed down from the elders to the young generation. According to the age structure of the vendors (Table 2), groups 31–60 and 61–90 at roughly equal in size. There was only a small group of young people in the market. Few young Miao appear to be trained in traditional knowledge and sustainable harvesting of medicinal plants, likely because most herbal materials are collected from wild plant populations, and there is small quantity for each plant. Compared to working in the urban areas, collecting and selling wild medicinal plants were only temporary job; it is less profitable. Even Miao medicine has a lot of growth potential, but for reasons of cost and time, it is hard for untrained people to develop a successful business. In interviews, most young people also expressed disbelief that studying indigenous knowledge can earn money for their life [18, 47]. Thus, in recent years a large number of rural young people have chosen to move to big cities to work and live. This phenomenon could have a negative influence on the inheritance and development of indigenous knowledge. It exposes the vulnerability of traditional medicinal knowledge if its transmission is limited by acculturation or inter-ethnic exchange from generation to generation [48].

### The names of Miao medicinal plants

The Miao often name medicinal plants according to their features such as color, morphology, usage, and flavor [49, 50], which is similar to the nomenclature of local people in Umnugobi Province, Mongolia [51]. There are three main types of nomenclature: (1) The word *jab*, which means medicine, is added to the medicinal plants. For example, the Miao name for *Epimedium acuminatum* is *jab ngol xid* which means “herbal medicine used to treat impotence.” Thus, this nomenclature can be formulated as “jab + usage”; (2) the used plant part is added to the name. For example, the Miao name for *Ophiopogon japonicus* is *zend nangx ngol yut*. These words mean tuber (*zend*), herb (*nangx*), and persistent cough (*ngol yut*). Thus, in Miao nomenclature *Ophiopogon japonicus* is clearly understood to be an herbaceous plant and its tubers can be used to treat persistent cough; (3) the Miao name for a medicinal plant may be adopted from the local dialect in the study area. For example, the Miao people’s name for *Bletilla striata* is *wul jut*, which is the local dialect name for this plant.

### How to protect the Miao people’s traditional medicine culture?

Nowadays, the Chinese government has recognized ethnomedicine and issued a series of policies to support their protection and development after the foundation of the whole country [52, 53]. However, it is still urgent to cultivate more professional talents in the field of ethnomedicine by issuing more preferential policies and funds.

Researchers from different agencies and enthusiasms are encouraged to strengthen the investigation of Miao medicine plants. Books and databases of medicinal plants can be published, with supports from foundations, and providing free access to local healers and those (especially young people) who are interested in Miao ethnomedicine. For species with significant economic value, scientific institutions should accelerate scientific research on artificial breeding and cultivation. The advanced theories and methods of pharmacology, chemistry, and molecular biology should be applied to study the traditional Miao medicinal knowledge and enhance Miao people's understanding and confidence. Because of its significance in economy and culture, the local government or administration agency may pay more attention to the medicinal market to provide a better environment for vendors and buyers. It is also necessary to encourage the Miao people to conserve medicinal plants in situ and ex situ, such as by planting endangered and preferred medicinal species in their home gardens or farmlands.

## Conclusion

This study shows that sociocultural customs related to medicinal plants have brought about their own unique influences on daily life and become indispensable components in the folk culture and social custom in Kaili. In this study, we analyzed the data collected from 116 vendors who sold fresh or dried herbal medicinal material of 237 plant species to treat a wide spectrum of illnesses and diseases. Most of these plants were used in the treatment of heat and detoxification, traumas, skin diseases, and wounds. Inflammatory diseases have the highest value of used citations, followed by stomach, intestine, and liver diseases. The occurrence of these diseases is likely associated with local living habits and environmental conditions. Three medicinal plant species, *Eleutherococcus nodiflorus, Sargentodoxa cuneata*, and *Stephania cepharantha*, which are used by the local people, have a particularly high public recognition and consistent patterns of use: The next step should include further studies on these plants’ chemistry, pharmacology, biological activity, and toxicity for potentially developing functional foods or pharmaceutical products.

Although high numbers of medicinal plant species have been reported to be used for human health problems, many wild species are being threatened by various anthropogenic factors, while conservation efforts are less practiced in the study area.

Furthermore, the knowledge on herbal remedies is held by elders, who are less educated, while most young people prefer to look for jobs in urban areas instead of studying traditional medicinal knowledge in the countryside. It is therefore urgent to find solutions for conserving and transmitting the traditional medicinal knowledge in the study area.

## Data Availability

All data generated or analyzed during this study are included in this published article.
